# Effects of heterozygous deletion of autism-related gene *Cullin-3* in mice

**DOI:** 10.1371/journal.pone.0283299

**Published:** 2023-07-10

**Authors:** Qiang-qiang Xia, Angela K. Walker, Chenghui Song, Jing Wang, Anju Singh, James A. Mobley, Zhong X. Xuan, Jeffrey D. Singer, Craig M. Powell

**Affiliations:** 1 Department of Neurobiology, University of Alabama at Birmingham Marnix E. Heersink School of Medicine, & Civitan International Research Center, Birmingham, AL, United States of America; 2 Department of Neurology, University of Texas Southwestern Medical Center, Dallas, TX, United States of America; 3 Department of Anesthesiology and Perioperative Medicine, University of Alabama at Birmingham Mass Spectrometry & Proteomics Shared Facility, Birmingham, AL, United States of America; 4 Department of Biology, Portland State University, Portland, OR, United States of America; University of Florida, UNITED STATES

## Abstract

Autism Spectrum Disorder (ASD) is a developmental disorder in which children display repetitive behavior, restricted range of interests, and atypical social interaction and communication. *CUL3*, coding for a Cullin family scaffold protein mediating assembly of ubiquitin ligase complexes through BTB domain substrate-recruiting adaptors, has been identified as a high-risk gene for autism. Although complete knockout of *Cul3* results in embryonic lethality, *Cul3* heterozygous mice have reduced CUL3 protein, demonstrate comparable body weight, and display minimal behavioral differences including decreased spatial object recognition memory. In measures of reciprocal social interaction, *Cul3* heterozygous mice behaved similarly to their wild-type littermates. In area CA1 of hippocampus, reduction of *Cul3* significantly increased mEPSC frequency but not amplitude nor baseline evoked synaptic transmission or paired-pulse ratio. Sholl and spine analysis data suggest there is a small yet significant difference in CA1 pyramidal neuron dendritic branching and stubby spine density. Unbiased proteomic analysis of *Cul3* heterozygous brain tissue revealed dysregulation of various cytoskeletal organization proteins, among others. Overall, our results suggest that *Cul3* heterozygous deletion impairs spatial object recognition memory, alters cytoskeletal organization proteins, but does not cause major hippocampal neuronal morphology, functional, or behavioral abnormalities in adult global *Cul3* heterozygous mice.

## Introduction

Autism Spectrum Disorder (ASD) is a developmental disorder in which children display atypical social interaction, communication, and repetitive behaviors/restricted interests and may struggle with a range of additional areas such as executive or intellectual functioning, communication, motor skills, reasoning/planning, and using language [1]. Due to its high prevalence of about 1 in 44 children in the United States, it is crucial to understand the underlying genetic basis for ASD. Naturally occurring *de novo* mutations that cause loss-of-function in the *Cul3* ubiquitin E3 ligase gene were identified in exome sequencing studies of people with autism [2–5].

Previously, we found that the loss of *Kctd13*, a gene in the 16p11.2 recurrent Copy Number Variant (CNV) region [6–15] encoding a binding partner of CUL3, reduces hippocampal synaptic transmission via the RhoA pathway [16], and decreases synapse numbers in the hippocampus, among other brain regions [16]. Interestingly, KCTD13 is an adaptor protein for CUL3, a ubiquitin ligase that has been characterized as a high-risk autism gene through genome-wide association studies [17]. Previous studies revealed that nonsense mutations in *CUL3* identified in ASD patients negatively regulate the physical interaction between the KCTD13 and CUL3 proteins [17]. RhoA is a regulator of the actin cytoskeleton and has recently been implicated in autism [18]. RhoA is also a target of KCTD13/CUL3 ubiquitination [19], however, we found that *Kctd13* deletion does not generate elevated RhoA until after P7 [16]. Because KCTD13 performs its function in part by binding to CUL3, we were interested in how *Cul3* deletion affects hippocampal synaptic function, behavior, and protein expression. We initially hypothesized that *Cul3* deletion may share common mechanisms with *Kctd13* deletion.

Deletion of *Cul3* results in lethality in homozygous offspring (WT: 40.3%, HET: 59.7%, HOM: 0 in 206 mice) [20], a much stronger phenotype than that in the *Kctd13* homozygous mouse model [16], suggesting additional and different mechanisms for *Cul3* deletion compared to *Kctd13* deletion. A few publications have examined *Cul3* heterozygous or conditional knockout genetic mouse models [21–24] and *Cul3* neurons derived from human inducible pluripotent stem cells [25], leading to a somewhat confusing literature regarding *Cul3* function in the brain.

In this study, we utilize a *Cul3* heterozygous deletion mouse model to characterize *Cul3*’s role in behavior, neuronal morphology, and neuronal/synaptic function. We find that *Cul3* heterozygous mice show minimal behavioral differences, minimal synaptic function differences in hippocampus, and minimal neuronal morphology differences in the hippocampus. Unbiased proteomics reveal changes in cytoskeletal organization proteins most prominently, among others.

## Materials and methods

### Animals

Floxed *Cul3* (f*Cul3*) mice were gifts from Jeffrey D. Singer Lab at Portland State University [26]. These f*Cul3* mice were originally made in a 129S1 genetic background (Jackson Labs Stock No: 002448) and then backcrossed to a C57BL/6J background for at least 6 or more generations. To create constitutive, germline *Cul3* heterozygous deletion mice, we bred Cul3^flox/+^ heterozygous mice with a cre-recombinase transgenic line Zp3-Cre 93Knw/J mice (Jackson Labs Stock No: 003651) that express cre-recombinase only in oocytes. These mice were then backcrossed to C57BL/6J wildtype males to generate multiple Cul3^flox/+^/Zp3-Cre+ breeders of both sexes. These Cul3^flox/+^/Zp3-Cre+ mice were bred together avoiding sibling matings, resulting in experimental mice being either Cul3^+/-^/Zp3-Cre+ mice or Cul3^+/+^/ZP3-Cre+ sex-matched, littermate pairs (“*Cul3* HET” and “WT”). It is important to note that male mice never express cre-recombinase; female mice also do not express cre-recombinase, though the oocyte does transiently express cre-recombinase prior to the completion of the first meiotic division. WT and *Cul3* HET mice were weighed periodically starting at 9 wks of age through 21 wks of age to examine postnatal growth trajectory.

Successful removal of *Cul3* gene exon 4–7 was identified by PCR using three primers as follows:

GACCACAACTTTCCTGATGAAGTACATGG (F);

GTGAGGCACATGATTAACACATGCATG (R);

TTCTACAAGGCACATGTGTGTGCATGTAC (R).

WT DNA produced a 329 bp band while KO DNA produced a 496 bp band.

### Ethics approval and consent to participate

All animal procedures were approved in writing in accordance with the guidelines of the Institutional Animal Care and Use Committee (IACUC) at the University of Alabama at Birmingham (UAB) following the US National Institutes of Health Guidelines for the Care and Use of Laboratory Animals.

### Antibodies

Antibodies were used at the following dilutions from the following companies: mouse anti-Cul3 (1:500, BD Biosciences, CAT#611848), rabbit anti-KCTD13 (1:1000, Atlas Antibodies, HPA043524), mouse anti-RhoA (1:500, Abnova, CAT#H00000387-M08), rabbit anti-Transgelin (1:500, Abcam, CAT#ab227566), rabbit anti-Tropomyosin-4 (1:1000, Abcam, CAT#ab181085), mouse anti-β-actin (1:1000, MP Biomedicals, CAT#691001) and mouse anti-α-tubulin (1:1000, Invitrogen, CAT# 13–8000). Secondary antibodies for Western blot were purchased from Thermo Scientific (goat anti-mouse-680, #35518 and goat anti-rabbit-800, #35571).

### Immunoblotting

**Fig 1:**
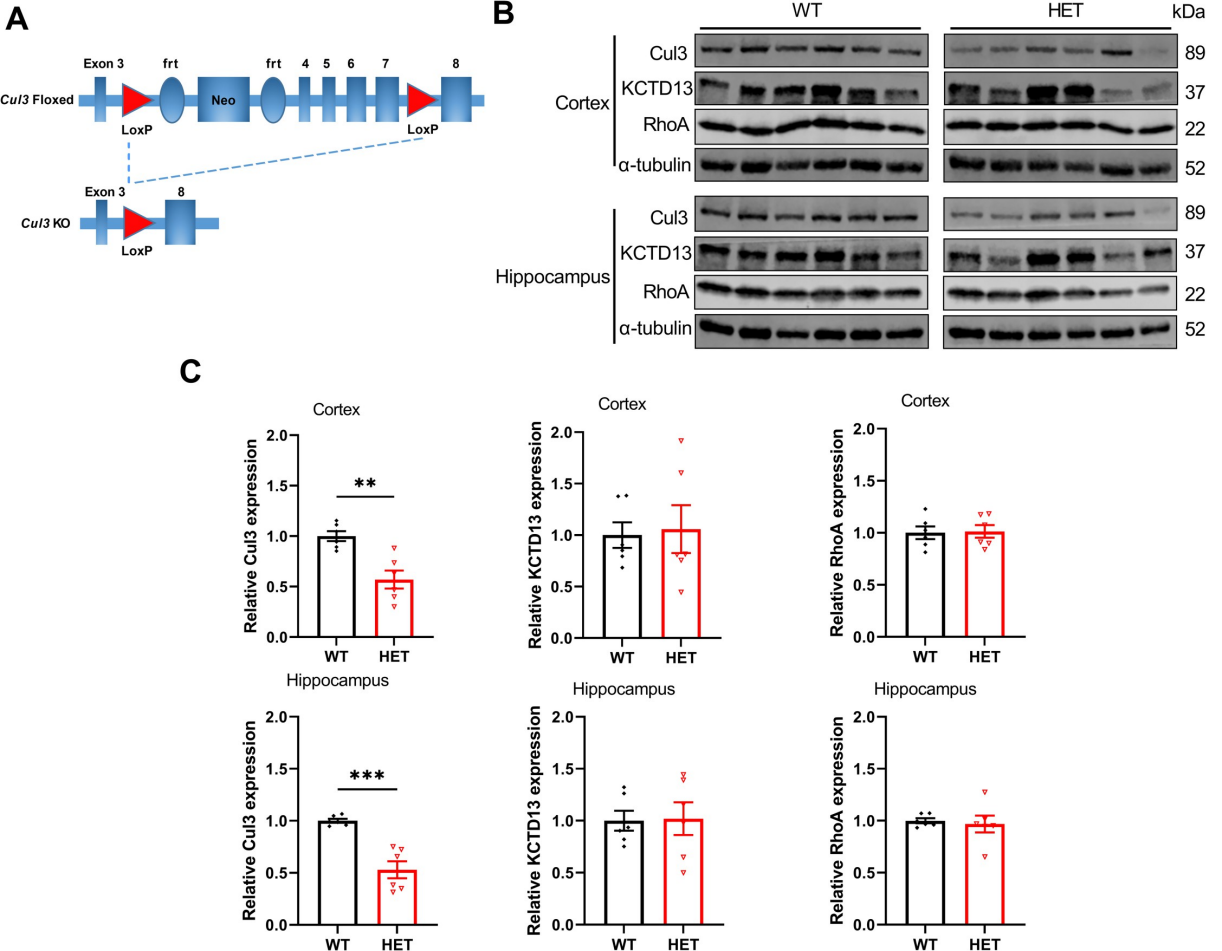
*Cul3* heterozygous deletion in *Cul3* global knockout mice. A) Diagram of *Cul3* global knockout mice model and Cre-LoxP knockout strategy. Exons 3–8 of the *Cul3* gene are represented along with LoxP, neomycin-resistance, and frt sites. Expression of Cre-recombinase in the germline results in recombination and loss of *Cul3* expression. B) Representative immunoblots of cortical and hippocampal homogenates for CUL3, KCTD13, RhoA and α-tubulin (WT N = 6 mice, male; HET N = 6 mice, male). C) Graphs of pooled data for Cul3, KCTD13, RhoA and α-tubulin. “HET” represents *Cul3* heterozygous deletion. Cul3 expression decreased to ∼56% in cortical neurons and decreased to ∼49% in hippocampal neurons respectively (F = 3.21, P = 0.0018 for Cul3 in cortex; F = 18.01, P = 0.0002 for Cul3 in hippocampus). Cul3 heterozygous deletion did not significantly alter KCTD13 or RhoA expression in cortex or hippocampus (F = 3.492, P = 0.8296 for KCTD13 in cortex; F = 2.671, P = 0.9163 for KCTD13 in hippocampus; F = 1.001, P = 0.8833 for RhoA in cortex, F = 11.16, P = 0.7174 for RhoA in hippocampus). *P<0.05, **P<0.01, ***P<0.001, unpaired student’s t-test; graphs depict mean ± SEM.

Crude lysates were collected in 2× Laemmli sample buffer (Bio-Rad, #1610737) supplemented with 2-Mercaptoethanol (Bio-Rad, #1610710) and boiled at 100°C for 5 min. Protein extracts were separated by SDS-PAGE and then transferred to a nitrocellulose membrane (Bio-Rad, Hercules, CA, United States). Membranes were blocked with Intercept Blocking Buffer (LI-COR, #927–60001, NE, United States) for 2 h at room temperature. Membranes were then incubated with the corresponding primary antibodies for 1 h at room temperature or 4°C overnight. The membranes were imaged on a LI-COR (Lincoln, NE, United States) Odyssey CLx Imaging System after incubating with corresponding secondary antibodies. α-tubulin or β-actin were used as internal controls with all protein levels normalized to the levels of these internal controls followed by normalizing all values to the average of the WT signals, allowing us to present SEMs for both WT and HET values (All original immunoblotting results from Figs 1B and 8G were included in S1 and S3 Figs).

### Microscopy, image processing, and analysis

Golgi staining was performed as previously described by using a FD Rapid Golgi staining kit [27]. Slides were visualized under a Zeiss LSM-800 Airyscan confocal microscope in the core facilities of University of Alabama at Birmingham Heersink School of Medicine. Images were captured with the same parameters and analyzed blind to genotype. The control and experimental groups were processed in parallel. The branching of Golgi-stained hippocampal neuron dendrites was measured and traced using Neurolucida 360 (MBF Bioscience) from coronal slices (150 μm). Spines on the secondary branches of apical dendrites in the CA1 region were counted. Spines were classified into four subtypes as previous described [28–30]: thin, stubby, mushroom and filopodia. Thin spines have a thin neck and a small bulbous head, and the diameters of neck and head are similar. Stubby spines typically have very short necks or do not have a neck. Mushroom spines have a thickened neck and a larger head than thin spines. Filopodia were long, thin dendritic membrane protrusions without a clear head and distinguished from other spines by having greater length. The classifying settings of Neurolucida 360 for spine subtypes were as following: head to neck ratio: 1.1; length to head ratio: 2.5; mushroom head size: 0.35 μm; filopodium length: 3 μm. If the ratio of the total length of the protrusion to the width of base of the protrusion was smaller than 2.5, the spine was classified as stubby; if the ratio was larger than 2.5, but the head diameter <0.35 μm, the spine was classified as thin. A mushroom spine was classified as a spine with a neck and a head diameter >0.35 μm. A filopodia spine was classified as a long, thin spine without a clear head and a total length smaller than 3 μm. Sholl analysis was based on the number of intersections per radius of shell. Numbers of dendrites, total dendritic length, and average dendritic length were made from confocal microscope images including all primary, secondary, tertiary, etc. dendrites using Neurolucida 360.

### Whole-cell patch-clamp recording

For miniature excitatory postsynaptic current (mEPSC) recording in hippocampus, horizontal slices (300 μm) containing dorsal hippocampus were prepared from 2–4 week old mice (n = 14 and n = 11 for WT and HET respectively, 5 male, 3 female, 17 sex-blind) of either sex. To record NMDA/AMPA current ratio in medial prefrontal cortex (mPFC), coronal slices (300 μm) containing prefrontal cortex (AP + 1.5–2.2) were prepared from 6–9 week old mice (n = 6 for both WT and HET; 7 male, 5 female) of either sex.

Slices were cut in ice-cold artificial cerebrospinal fluid (aCSF, containing in mM: 125 NaCl, 2.5 KCl, 1.25 NaH_2_PO_4_, 2 CaCl_2_, 1 MgCl_2_, 25 NaHCO_3_, 10 glucose, pH 7.5, bubbled with 95% O_2_/5% CO_2_) using a vibrating tissue slicer (VT1000, Leica). Slices were transferred to a holding chamber where they remained in oxygenated aCSF at 34°C until use. During recording, slices were transferred to a submerged recording chamber where they were continuously perfused at 2 ml/min with oxygenated aCSF and maintained at 34 ± 2°C using an inline temperature controller. Neurons were visualized with infrared differential video interference microscopy (IR-DIC). Whole-cell recordings were obtained with standard-wall borosilicate glass pipettes (2–5 MΩ) filled with the following solution (in mM): 110 CsMeSO_3_, 15 CsCl, 8 NaCl, 10 HEPES, 2 EGTA, 10 TEA, 2 Na-ATP, 0.3 Na_2_GTP, pH 7.3 and osmolality of 285 mOsmol.

Resting membrane potential (RMP) was obtained by switching to current-clamp immediately after whole-cell mode was established. Access resistance (R_a_) was monitored throughout the experiments and recordings were terminated if the change of R_a_ was > 20%. Membrane capacitance (Cm) and resistance were obtained from pCLAMP 10 software (Molecular Devices) by application of a depolarizing pulse of 5 mV. For mEPSC recording, tetrodotoxin (TTX, 1 μM) and picrotoxin (100 μm) were added to the perfusion solution and spontaneous activities were recorded for 10 min and analyzed between 5–10 min after whole-cell mode was obtained. For NMDA/AMPA current ratio recording in the mPFC, picrotoxin (100 μm) was added to the perfusion solution. Evoked EPSCs in layer 5 pyramidal neurons were evoked using a concentric bipolar stimulating electrode positioned in layer 2/3. AMPAR-mediated evoked EPSCs (eEPSCs) were recorded at −70 mV and measured as the peak response following the stimulus. NMDAR-mediated EPSCs were recorded at +40 mV and measured as the mean current over a 5-ms window, 50 ms after the stimulus. Mean EPSCs were an average of 15 sweeps obtained at 0.1 Hz.

All recordings were obtained with a MultiClamp 700B amplifier system (Molecular Devices, Union City, CA). Experiments were controlled by pCLAMP 10 software (Molecular Devices), and the data were acquired with the Digidata 1440A acquisition system (Molecular Devices). Recordings were filtered at 4 kHz and digitized at 50–100 kHz.

### Field recording

Field recording was performed in an interface chamber (Fine Science Tools, Foster City, CA) on dorsal hippocampal slices from 6–7 week old mice (male WT = 5, HET = 4; female, WT = 4, HET = 3). Oxygenated ACSF (95%/5% O_2_/CO_2_) was warmed (30° C, TC-324B temperature controller, Warner Instruments, Hamden, CT) and perfused into the recording chamber at a rate of 1 mL/min. fEPSPs were recorded in stratum radiatum with an ACSF-filled glass recording electrode (1–3 M). Extracellular stimuli were administered (Model 2200 stimulus isolator, A-M Systems) on the border of area CA3 and CA1 along the Schaffer-collaterals using handmade, formvar-insulated nichrome wire (A-M system, Cat# 761000) electrodes. The experimental stimuli were set at 50% of the maximum fEPSP, which is obtained from input/output curves generated via 1mV current steps from 4 to 15mV with a stimulation frequency of 0.05 Hz. The stimulus intensity remained consistent in all subsequent experiment. Paired-pulse ratio was measured at various interstimulus intervals (10, 20, 50, 100, 150, 200, 250, 300 msec). High-frequency stimulus-induced LTP was induced by theta burst stimulation (tbs), which includes 3 trains of stimuli at the test stimulus intensity with 20s intervals. Each train is composed of 10 stimulus bursts delivered at 5Hz, with each burst consisting of four pulses delivered at 100Hz. Synaptic efficacy was monitored 20 min prior to and 1 h following induction of LTP by recording fEPSPs every 20 sec (traces were averaged for every 2 min interval). Fiber volley amplitude and initial fEPSP slope were measured for data analysis.

### Behavioral overview

All mice used for behavioral testing were age- and sex-matched littermate progeny of heterozygous *Cul3* mutant crossings as described above in detail. An experimenter blind to genotypes performed all behavioral tests. The behavioral cohort consisted of the following sex- and age-matched littermate pairs: WT = 20, HET = 20 (male WT = 10, HET = 10; female WT = 10, HET = 10) for elevated plus maze, dark/light, open field, locomotor habituation, social approach of caged conspecific, genotype/sex-matched reciprocal social interaction, olfactory food finding, grooming, marble burying and pre-pulse inhibition tests; WT = 20, HET = 19 (male WT = 10, HET = 9; female WT = 10, HET = 10) were used for rotarod, hot plate, olfactory preference for social odors and novel and spatial object recognition test. Mice were 4–9 weeks apart, with the youngest age being 10 weeks at onset of testing. At least 2 days up to one week have been allowed for animals to recover following each behavioral task. Behavioral tests were done in the order in which they appear in the methods below. The behavioral tests were conducted in an order with less stressful tests at the start of the battery and more stressful tests at the end of the battery.

#### Elevated plus maze

Mice were placed in the center of the maze (open arms 30 × 5 cm; closed arms 30 × 5 × 25 cm) and allowed to freely explore the maze for 5 min. The light was set to approximately 7 lux. Noldus Ethovision version 3.1 was used to track and record mouse behavior [31].

#### Dark/light test

Animals were placed in the dark chamber of light/dark test apparatus with a bright fluorescent light placed directly over the top of the light chamber (light chamber 25 × 26 cm, 2,066 lux; dark chamber 25 × 26 cm, ∼1 lux) and allowed to habituate for 2 min. After habituation, mice were allowed to explore both chambers freely for 10 min [31].

#### Open field

Mice were placed in one of the corners of an open arena (44 × 44 × 44 cm, ∼7 lux) and allowed to freely explore for 10 min. Time spent in the center of the arena (15 × 15 cm) and locomotor activity were measured. The behavior was recorded and tracked by CleverSys TopScan software [32].

#### Locomotor habituation

Mice were placed in clean home cages (27.3 × 16.5 × 12.7 cm) with 1 in of bedding and allowed to explore freely for 2 h under red lighting. Horizontal locomotor activity (that is, the number of photobeam breaks) was measured by computer software (San Diego Instruments). Data were analyzed and plotted in 5-min bins [31].

#### Caged conspecific test

Mice were placed into the open field arena (44 x 44 x 44 cm, ∼7 lux) containing a small, rectangular empty cage with holes in it for olfactory cue transmission. The experimental mouse explored the arena for 5 min (trial 1). During the second 5-min trial, a novel, sex-matched target mouse was placed in the small rectangular cage as a “social target”. Interaction of the mouse with the social target was tracked using CleverSys ObjectScan Software [16].

#### Reciprocal social interaction

Mice of the same sex and genotype were placed into the open field arena (44 × 44 × 44 cm, ∼7 lux). The mice were allowed to explore the arena for 5 min. Combined reciprocal social interaction of the two mice was tracked and scored in an automated fashion by CleverSys SocialScan Software [16]. Social interaction was tracked and scored by CleverSys SocialScan Software using the intrinsic parameters programmed into the software. The system will objectively determine the social interactions of approach, avoidance, sniffing, and contact based on the automatically detected heads and tails of animals, and their relative spatial positions and movements.

#### Olfactory food finding

A peanut butter cookie was placed in the middle of a clean cage 1 inch below the surface of a thick layer (∼10 cm) of bedding. Mice were placed in the cages and were allowed free exploration for 10 min. The test stopped when the mouse uncovered the cookie [16].

#### Grooming

Mice were placed in empty cages where they were habituated for 10 min followed by a 10-min scoring period by 2 observers blind to genotype for time spent in any grooming behavior (grooming the face, head, body, or tail). Scoring was from videotape [32]. When self-grooming, a mouse is cleaning its own body and fur by licking and scratching. Grooming consists of syntaxes that are small (paw lick) to mid-size movements (unilateral and bilateral face wash) and large movements (flank licking). There are also rare syntaxes such as genital and tail grooming [33].

#### Marble burying

Twenty marbles were evenly placed in a novel home cage with 5 cm of bedding. Mice were given 30 min to explore the cage in a well-lit room (∼70 lux). After 30 min, the number of marbles buried was recorded. A marble was defined as buried when less than one-third of the marble was visible [32].

#### Rotarod

Mice were placed on a stationary rotarod (IITC Life Science). After the test began, the rod accelerated from 0 to 45 r.p.m. over 5 min. The latency to fall off the rod was measured if a mouse fell off the rod or held onto the rotating rod for two complete revolutions. Each mouse received four trials per day for 2 days [31].

#### Novel and spatial object recognition

Briefly, mice were habituated for 4 days to an open arena (44 × 44 × 44 cm, ∼7 lux) with spatial cues attached to the inside of arena walls. Each mouse underwent one 5 min trial per day for 4 days of habituation. On the fifth day (test day), all animals received seven 5-min trials each with 6- or 45-min intervals. For the fifth trial, three identical objects were placed in the arena. Each object was approximately 12.5 cm from the closest wall. During the sixth trial (spatial test), one of the objects (object A) was relocated to the opposite corner. During the seventh trial (novel object test) a small statue replaced object B. The behavior was recorded using CleverSys ObjectScan [16].

#### Hotplate sensitivity

Mice were placed on a black, anodized plate that was held at a constant temperature of 52°C (IITC Life Sciences model 39 hotplate) covered with a Plexiglas enclosure. Mice were removed after the first hindpaw lick or after 30 s if no response was elicited [34].

#### Olfactory preference for social odors

Initially, mice were placed in an open arena (44 × 44 × 44 cm, ∼7 lux) for 5-min recording with a slide containing a nonsocial smell (rubbed with distilled water). Immediately after, mice were allowed to interact with a slide containing a “social” smell (slide rubbed on the anogenital region of an unfamiliar C57BL/6J WT mouse) for another 5-min recording. The box was wiped with 70% ethanol and air dried between mice [34].

#### Prepulse inhibition and startle

Both prepulse inhibition and startle response were conducted as previously described [35]. Briefly, a cylinder mounted on a piezoelectric accelerometer detected and transduced mouse movements and were placed in sound attenuation chambers (San Diego Instruments, San Diego, CA, USA). White noise at various volumes (dB) was delivered by speakers mounted ∼33cm above each cylindrical mouse enclosure. Mice were then exposed to stimuli ranging from 0 to 120 dB, measuring the startle response amplitude in arbitrary units. For prepulse inhibition (PPI) experiments, mice were given five types of trials over a 22-min session: pulse alone (40 msec, 120 dB, white noise pulse), three different prepulse/pulse trials (20 msec prepulse of 4, 8, or 16 dB above background noise level of 70 dB preceded the 120 dB pulse by 100 msec onset–onset interval), and no-stimulus. Pseudorandom presentation of trials with an average of 15 sec (7–23 sec) between the trials avoided habituation. Mice were habituated to the apparatus for 5 min, followed by four blocks of test trials. The first and last blocks consisted of six “pulse-alone” trials. Blocks 2 and 3 each contained six “pulse-alone” trials, five of each level of prepulse/pulse trials, and five no-stimulus trials.

### Proteomics analysis

#### Sample preparation

Global proteomic analysis was performed on the hippocampal brain region of adult WT and *Cul3* HET, 8–10 weeks old mice. Proteomics analysis was carried out as previously referenced [36] with minor changes (under section 2.5 nLC-ESI-MS2 under Protein IDs for GeLC). All proteins extracts were obtained using T-PER™ Mammalian Protein Extraction Reagent (Thermo Fisher Scientific, Cat. # 78510) containing Halt™ Protease Inhibitor Cocktail (Thermo Fisher Scientific, Cat.# 78429) and Dounce-homogenized in lysis buffer, 20–30 strokes per sample, and centrifuged (∼12K(g), 10min.@ 4°C) to remove debris. Protein extracts were then quantified using Pierce BCA Protein Assay Kit (Thermo Fisher Scientific, Cat. # PI23225). We applied 40 μg of protein per sample diluted to 35 μL using NuPAGE LDS sample buffer (1x final conc., Invitrogen, Cat. # NP0007). Proteins were then reduced with DTT and denatured at 70°C for 10 min prior to loading onto Novex NuPAGE 10% Bis-Tris Protein gels (Invitrogen, Cat.# NP0315BOX) and separated (35 min at 200 constant V). The gels were stained overnight with Novex Colloidal Blue Staining kit (Invitrogen, Cat. # LC6025). Following de-staining, each lane was cut into 6-MW fractions and equilibrated in 100 mM ammonium bicarbonate (AmBc); each gel plug was then digested overnight with Trypsin Gold, Mass Spectrometry Grade (Promega, Cat. # V5280) following manufacturer’s instructions. Peptide extracts were reconstituted in 0.1% Formic Acid/ ddH_2_O at 0.1μg/μL.

#### Mass spectrometry

Peptide digests (8 μL each) were injected onto a 1260 Infinity nHPLC stack (Agilent Technologies), and separated using a 75 μm inside diameter x 15 cm pulled tip C-18 column (Jupiter C-18 300 Å, 5 micron, Phenomenex). This system runs in-line with a Thermo Orbitrap Velos Pro hybrid mass spectrometer, equipped with a Nanospray Flex^TM^ ion source (Thermo Fisher Scientific); all data were collected in collision-induced dissociation mode. The nano-High Performance Liquid Chromatography is configured with binary mobile phases that includes solvent A (0.1%FA in ddH_2_O), and solvent B (0.1%FA in 15% ddH_2_O / 85% ACN), programmed as follows; 10min @ 5%B (2μL/ min, load), 90min @ 5%-40%B (linear: 0.5nL/ min, analyze), 5min @ 70%B (2μL/ min, wash), 10min @ 0%B (2μL/ min, equilibrate). Following each parent ion scan (300-1200m/z @ 60k resolution), fragmentation data (MS2) were collected on the top most intense 15 ions. For data dependent scans, charge state screening and dynamic exclusion were enabled with a repeat count of 2, repeat duration of 30 s, and exclusion duration of 90 s.

#### MS data conversion and searches

The XCalibur RAW files were collected in profile mode, centroided and converted to MzXML using ReAdW v. 3.5.1. The mgf files were then created using MzXML2Search (included in TPP v. 3.5) for all scans. The data was searched using SEQUEST (Thermo Fisher Scientific), which was set for three maximum missed cleavages, a precursor mass window of 20 ppm, trypsin digestion, variable modification C @ 57.0293, and M @ 15.9949 as a base setting. Searches were performed with a cleaned up version (redundant sequences removed) of the *Mus Musculus* specific subset of the UniProt100 database.

#### Peptide filtering, grouping, and quantification

The list of peptide identifications (IDs) generated based on SEQUEST search results were filtered using Scaffold (Protein Sciences, Portland Oregon). Scaffold filters and groups all peptides to generate and retain only high confidence IDs while also generating normalized spectral counts (N-SC’s) across all samples for the purpose of relative quantification. The filter cut-off values were with a minimum peptide length of >5 amino acids, with no MH+1 charge states, with peptide probabilities of >80% confidence interval, and with the number of peptides per protein ≥2. The protein probabilities were set to a >99.0% confidence interval, and a false discovery rate (FDR) <1.0. Scaffold incorporates the two most common methods for statistical validation of large proteome datasets, the FDR and protein probability [37–39]. Relative quantification across experiments were then performed via spectral counting [40, 41], in this case, spectral count abundances were then normalized between samples [42].

#### Statistical and multivariate analysis

For the proteomic data generated, a statistical analysis was performed between each pair-wise comparison, combined with t-test (single tail, unequal variance, cut off of p < 0.05). For protein abundance ratios determined with normalized spectral counts (NSCs), we set a 1.5-fold change as the threshold for significance, determined empirically by analyzing the inner-quartile data from the control experiments using ln-ln plots, where the Pierson’s correlation coefficient (R) is 0.98, and >95–99% of the normalized intensities fell between the set fold change. In each case, both tests (T-test and fold change) have to pass in order to be considered significant. All multivariate analysis, including 2D HCA HeatMaps, PCA plots, etc., were carried out using Qlucore Omics Explorer (Qlucore, Lund Sweden).

#### Systems analysis

Gene ontology assignments and pathway analysis were carried out using MetaCore (GeneGO Inc., St. Joseph, MI). Interactions identified within MetaCore are manually correlated using full text articles. Detailed algorithms have been described previously [43, 44].

### Statistics

Statistics were carried out using Graphpad Prism 9.0.2 (San Diego, CA, United States). Data were subjected to D’Agostino & Pearson test of normality. Data sets that exhibited a normal distribution were analyzed with unpaired parametric student’s t-test; those data sets that did not exhibit a normal distribution were analyzed using non-parametric statistical analysis with Kruskal–Wallis tests to compare more than two groups and Mann–Whitney tests to compare two groups. For Sholl analysis, 30 neurons in total from 10 separately derived mouse hippocampi were analyzed. For spine density analysis, 50 neurons in total from 10 separately derived mice hippocampi were analyzed. For electrophysiological analysis, data were analyzed from individual cells. Unpaired parametric Student’s t-test was used for biochemistry analysis, spine density analysis, whole-cell patch-clamp recording analysis, behavioral analysis and proteomic analysis to compare means between genotypes. For Sholl analysis, field recording and behavioral analysis, we performed either 2-way analyses of variance (ANOVA) or 3-way repeated measures (ANOVA) with genotype and sex as the main variables and repeated measures when appropriate. A Newman–Keuls post hoc test was applied to determine significant effects and interactions. Experimenters were blind to genotype or treatment for all comparisons. *P<0.05, **P<0.01, ***P<0.001, graphs depict mean ± SEM (Standard Error of the Mean).

## Results

### *Cul3* heterozygous deletion in *Cul3* global knockout mice

We used the Cre-loxP system and germline cre recombinase expression to knockout *Cul3* (Figs 1A and S1); f*Cul3* mice were crossed with Zp3-cre 93Knw transgenic mice, whose cre recombinase is expressed in oocytes transiently, to generate *Cul3* global knockout mice. The complete knockout of *Cul3* results in lethality in our hands and other’s [20, 45], so in this study we examined only *Cul3* HET mice and WT littermates as controls. We first verified Cul3 protein level by immunoblots that were first normalized to α-tubulin and then normalized to the average of the WT level (Figs 1B and S1); this allows one to show standard error of the mean (SEM) in the WT values (error bars). We found that the normalized Cul3 expression in *Cul3* heterozygous cortex was reduced to ∼57%, and in hippocampus it was reduced to ∼53% of the WT control level respectively (Figs 1C and S1). KCTD13 and RhoA expression level was comparable between *Cul3* HET and WT mice in both cortex and hippocampus (Figs 1C and S1), a surprising result given that RhoA levels were found to be significantly elevated in *Kctd13* deletion mice [16] and that KCTD13 is a binding partner of Cul3.

### *Cul3* heterozygous deletion does not affect general measures of growth, motor, or sensory abilities

**Fig 2:**
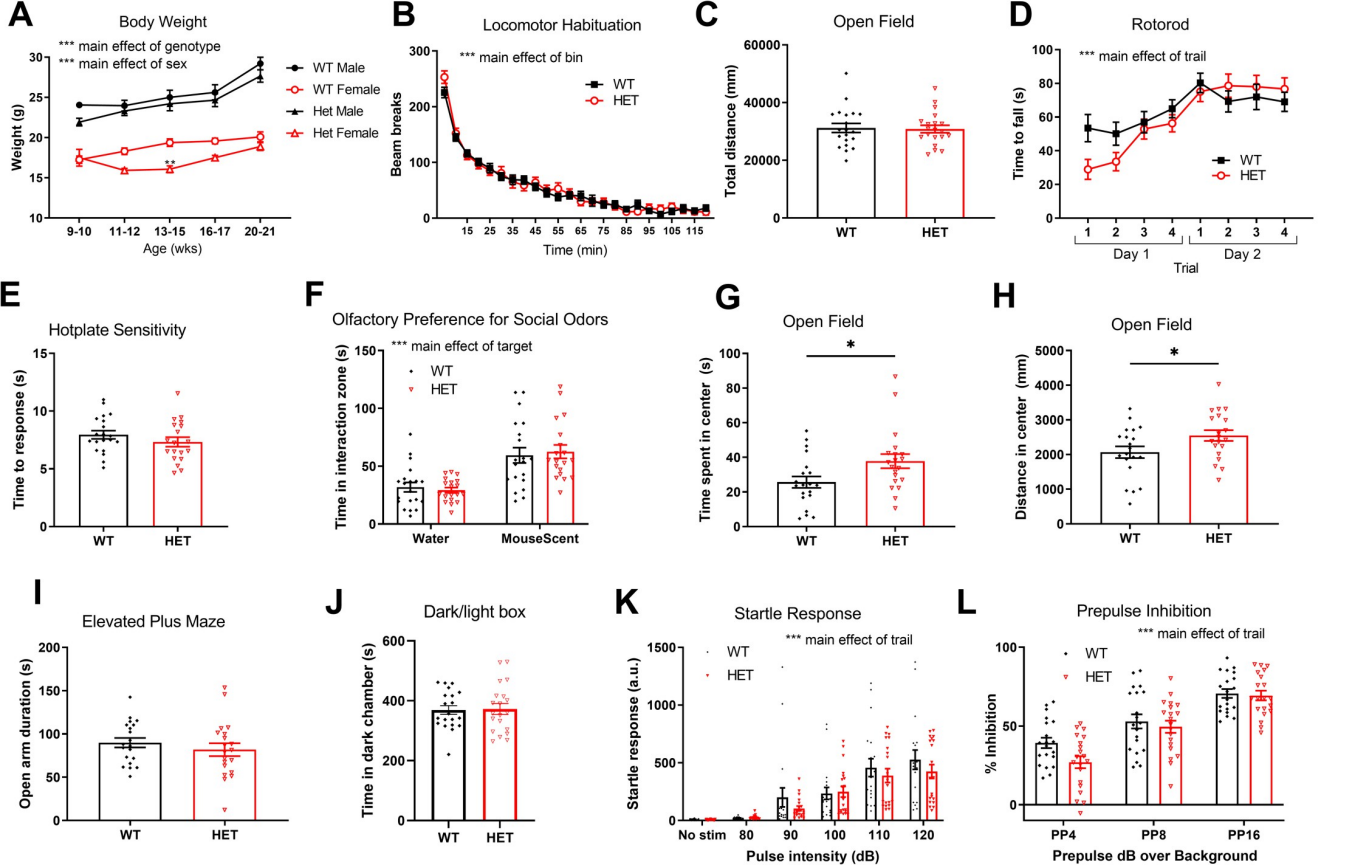
Body weight, motor skills, sensory ability, and anxiety-like behavior in *Cul3* global knockout mice. A) Body weights measured periodically over time do not differ substantially between *Cul3* HETs and WT littermates, a main effect of sex or genotype was observed; P < 0.0001 (9–10 wks, WT = 19, HET = 10 (male, WT = 10, HET = 7; female, WT = 9, HET = 3) , P = 0.103 for male, P = 0.9974 for female. 11–12 wks, WT = 15, HET = 11 (male, WT = 10, HET = 6; female, WT = 5, HET = 5) , P = 0.9009 for male, P = 0.1927 for female. 13–15 wks, WT = 18, HET = 13 (male, WT = 9, HET = 7; female, WT = 9, HET = 6), P = 0.8276 for male, P = 0.0058 for female. 16–17 wks, WT = 20, HET = 19 (male, WT = 10, HET = 9; female, WT = 10, HET = 10), P = 0.6733 for male, P = 0.0686 for female. 20–21 wks, WT = 20, HET = 20 (male, WT = 10, HET = 10; female, WT = 10, HET = 10), P = 0.258 for male, P = 0.4851 for female). B) Locomotor activity measured over 2 h in clean mouse cages using beam break counts was unchanged in *Cul3* HETs vs. WT littermates, a main effect of bin was observed; P < 0.0001 (P = 0.5895, male, WT = 10, HET = 10; female, WT = 10, HET = 10). C) No difference in total distance travelled in an open field arena (F = 1.499, P = 0.8636, male, WT = 10, HET = 10; female, WT = 10, HET = 10). D) No significant difference in time to fall from the accelerating rotarod was observed between *Cul3* HETs and WT littermates. A main effect of trail was observed; P < 0.0001 (P = 0.159, male WT = 10, HET = 9; female, WT = 10, HET = 10). E) No difference in time to paw-lick response on the hotplate sensory task between *Cul3* HETs and WT littermates (F = 1.28, P = 0.2635, male, WT = 10, HET = 9; female, WT = 10, HET = 10). F) Time spent in the mouse scent interaction zone was unchanged between *Cul3* HETs and WT littermates. A main effect of target was observed; P < 0.0001 (P = 0.9194 for water, P = 0.883 for mouse scent, male, WT = 10, HET = 9; female, WT = 10, HET = 10). G) Time spent in center zone of an open field arena is increased in *Cul3* HETs vs. WT littermates (F = 1.533, P = 0.0283, male, WT = 10, HET = 10; female, WT = 10, HET = 10). H) Distance travelled in center zone of an open field arena is increased in *Cul3* HETs vs. WT littermates (F = 1.177, P = 0.042, male, WT = 10, HET = 10; female, WT = 10, HET = 10). I) No change in time spent in open arms of elevated plus maze (F = 1.818, P = 0.3832, male, WT = 10, HET = 10; female, WT = 10, HET = 10). J) No change in time spent in dark chamber in dark/light test (P = 0.8892 for time in dark chamber, male, WT = 10, HET = 10; female, WT = 10, HET = 10). K) Startle response in arbitrary units in response to white noise auditory stimulus pulse of various volumes in decibels (dB). A main effect of trail was observed; P < 0.0001 (P>0.9999 for no stimulus group, P>0.9999 for 80 dB group, P = 0.7174 for 90 dB group, P>0.9999 for 100 dB group, P = 0.9423 for 110 dB group, P = 0.6434 for 120 dB group, male, WT = 10, HET = 10; female, WT = 10, HET = 10). L) Prepulse inhibition of startle (%) as a function of the prepulse volume above background noise (4, 8, & 16 dB above background). A main effect of trail was observed; P < 0.0001 (P = 0.0531 for PP4 group, P>0.9999 for PP8 group, P>0.9999 for PP16 group, male, WT = 10, HET = 10; female, WT = 10, HET = 10). *P<0.05, **P<0.01, ***P<0.001, unpaired student’s t-test, 2-way ANOVA or 3-way ANOVA test, compared between genotypes; graphs depict mean ± SEM. (See S1 Table in S1 File for detailed statistics).

WT and *Cul3* HET mice were weighed periodically between 9–21 wks of age to examine their growth trajectory. Males and females are plotted separately due to the known difference in weight between sexes in mice. *Cul3* HET mice exhibited comparable body weight compared to WT littermate mice with the exception of decreased weight in female *Cul3* HETs at a single timepoint (Fig 2A). No significant differences in locomotor activity were observed over 2 h in clean mouse cages (Fig 2B) or over 10 min in an open field arena (Fig 2C). Motor coordination on the accelerating rotarod was also not significantly different in *Cul3* HETs compared to WT littermates (Fig 2D). The initial coordination on the first trial of the first day of rotarod testing was significantly decreased in HET mice when tested using an unpaired t-test with the first trial alone, suggesting decreased initial motor coordination with intact motor learning; in the more appropriate ANOVA analysis of the entire rotarod experiment, however, the initial trial did not quite reach statistical significance (Fig 2D). Time to respond in the hotplate assay was comparable in both groups (Fig 2E). In a crude assay of olfaction, both *Cul3* HETs and WT littermates demonstrated a preference for a slide with mouse scent over a slide with no scent (Fig 2F). Both time spent and distance travelled in center zone of an open field arena significantly increased in Cul3 HETs compared to those in WT littermates (Fig 2G and 2H). No significant change was found in time spent in open arms of elevated plus maze and in dark chamber in dark/light test (Fig 2I and 2J). Startle responses to varying auditory pulses demonstrated no differences in startle response amplitudes at each level, suggesting that both the startle responses and auditory threshold to elicit a startle response are similar in each group (Fig 2K). Prepulse inhibition, a measure of sensorimotor gating, was unchanged in *Cul3* HETs compared to WT littermates (Fig 2L). These findings demonstrate largely intact sensory, motor, and anxiety-like behaviors in the *Cul3* heterozygous mutants.

### *Cul3* heterozygous deletion does not alter repetitive behavior or social interaction

**Fig 3:**
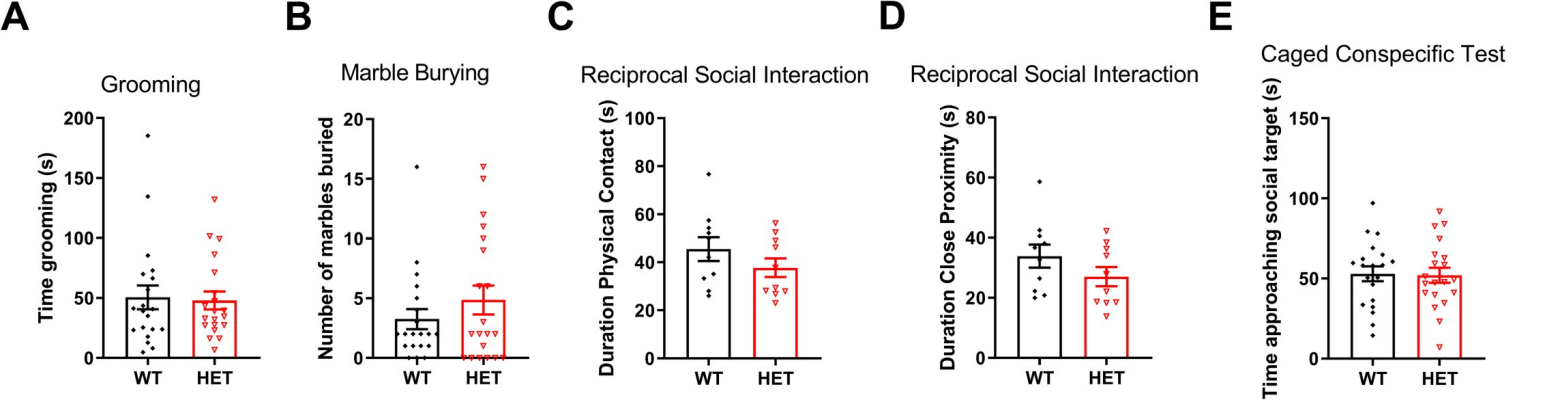
Repetitive behaviors and social interaction and approach. A) No difference in time spent grooming between *Cul3* HETs and WT littermates (F = 1.778, P = 0.8323, male, WT = 10, HET = 10; female, WT = 10, HET = 10). B) No difference in number of marbles buried in a marble burying task (F = 2.041, P = 0.2847, male, WT = 10, HET = 10; female, WT = 10, HET = 10). C) No difference in time spent in direct physical contact during a reciprocal social interaction task examining sex-matched WT/WT pairs and HET/HET pairs of mice in an open arena (F = 1.633, P = 0.2316, male, WT = 5 pairs, HET = 5 pairs; female, WT = 5 pairs, HET = 5 pairs). D) No difference in time spent in close proximity during a reciprocal social interaction task of genotype and sex-matched WT/WT and HET/HET pairs (F = 1.438, P = 0.1863, male, WT = 5 pairs, HET = 5 pairs; female, WT = 5 pairs, HET = 5 pairs). E) No difference in time spent in proximity (approaching) a caged social target in an open arena (F = 1.035, P = 0.8911, male, WT = 10, HET = 10; female, WT = 10, HET = 10). *P<0.05, **P<0.01, ***P<0.001, unpaired student’s t-test; graphs depict mean ± SEM.

No significant differences were found in time spent grooming (Fig 3A) or number of marbles buried (Fig 3B), indicating no significant change in repetitive behaviors. Reciprocal social interaction between sex-matched and genotype-matched mice was also not significantly changed including direct physical contact (Fig 3C) and time spent in close proximity (Fig 3D). Time spent approaching a caged social target in an open arena was comparable between *Cul3* HETs and WT littermates (Fig 3E).

### *Cul3* heterozygous deletion alters spatial object recognition memory

**Fig 4:**
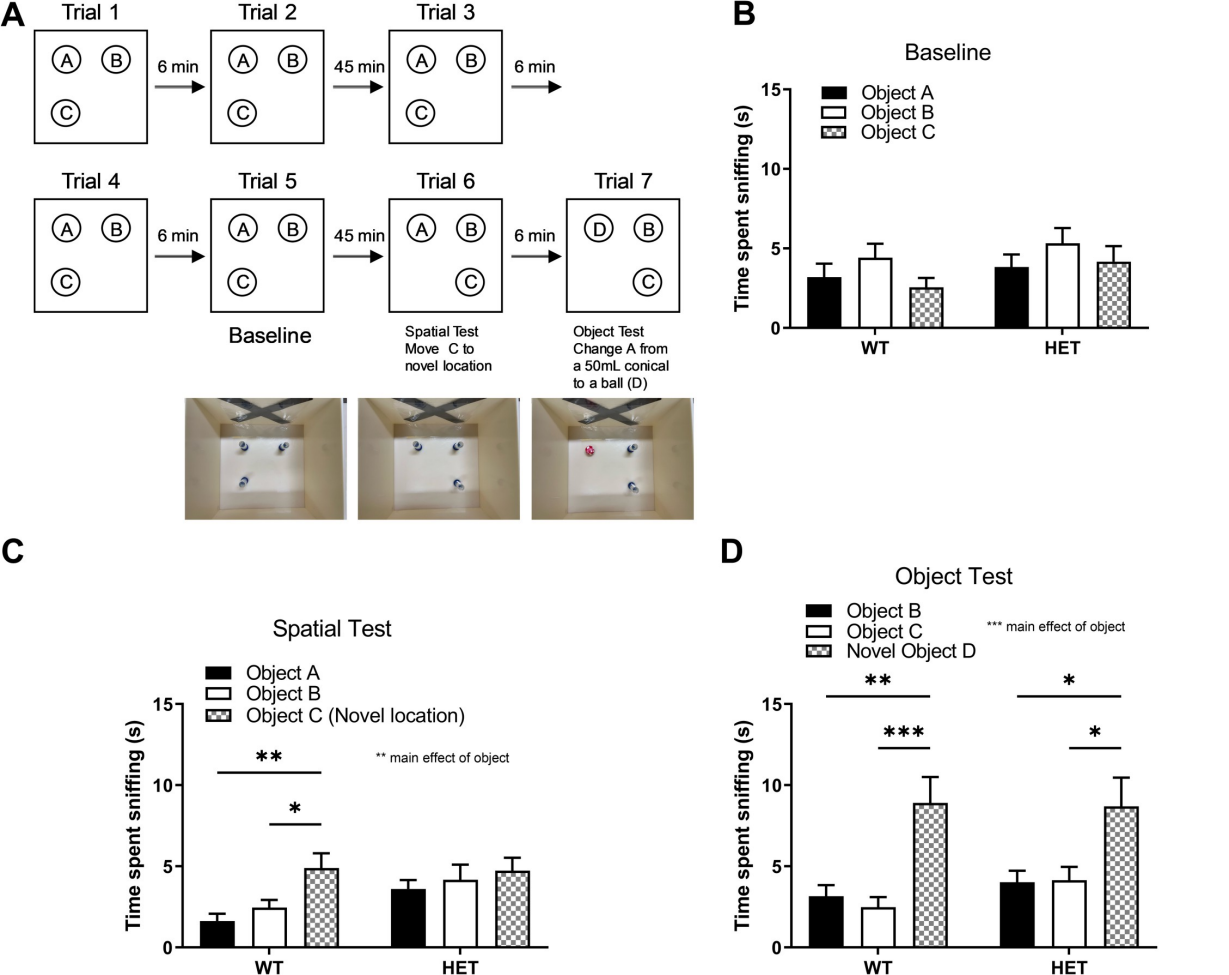
Spatial object recognition and novel object recognition tasks. A) Experimental schematic of the sequential Spatial Object and Novel Object recognition tasks. B) Initial time spent interacting with objects during baseline habituation is equivalent for *Cul3* HETs and WT littermates (P = 0.1533). C) WT mice exhibit spatial object recognition learning by spending significantly more time with the object (Obj C) in a new spatial location compared to objects in same location as before. A main effect of object was observed; P = 0.0092 (P = 0.0048 for Obj A vs. Obj C, P = 0.05 for Obj B vs. Obj C). *Cul3* HETs show no spatial object recognition learning by showing no preference for the newly localized object (P = 0.6016 for Obj A vs. Obj C, P = 0.9245 for Obj B vs. Obj C). D) *Cul3* HETs and WT littermates both demonstrate significant novel object recognition with significantly more time spent with the novel object compared to the two familiar objects (P<0.0001). A main effect of object was observed; P<0.0001. Male, WT = 9, HET = 9; female, WT = 10, HET = 10. *P<0.05, **P<0.01, ***P<0.001, 2-way ANOVA with Tukey’s post-hoc analyses; graphs depict mean ± SEM.

Sequential spatial object recognition and novel object recognition tasks were performed as in the schematic outlined in Fig 4A. Initial time spent interacting with objects during baseline habituation was comparable for *Cul3* HETs and WT littermates (Fig 4B). WT mice exhibited spatial object recognition learning by spending significantly more time interacting with the object in a new spatial location compared to objects in same location as before. *Cul3* HETs showed no significant preference for the newly localized object (Fig 4C). Both *Cul3* HETs and WT littermates demonstrated significant novel object recognition with significantly more time spent with the novel object compared to the two familiar objects (Fig 4D). These data demonstrate that *Cul3* HETs perform normally in novel object recognition but exhibit subtle but significant differences in spatial objection recognition.

### *Cul3* heterozygous deletion slightly affects dendritic complexity, but not dendrite length, dendrite branching, or spine density

**Fig 5:**
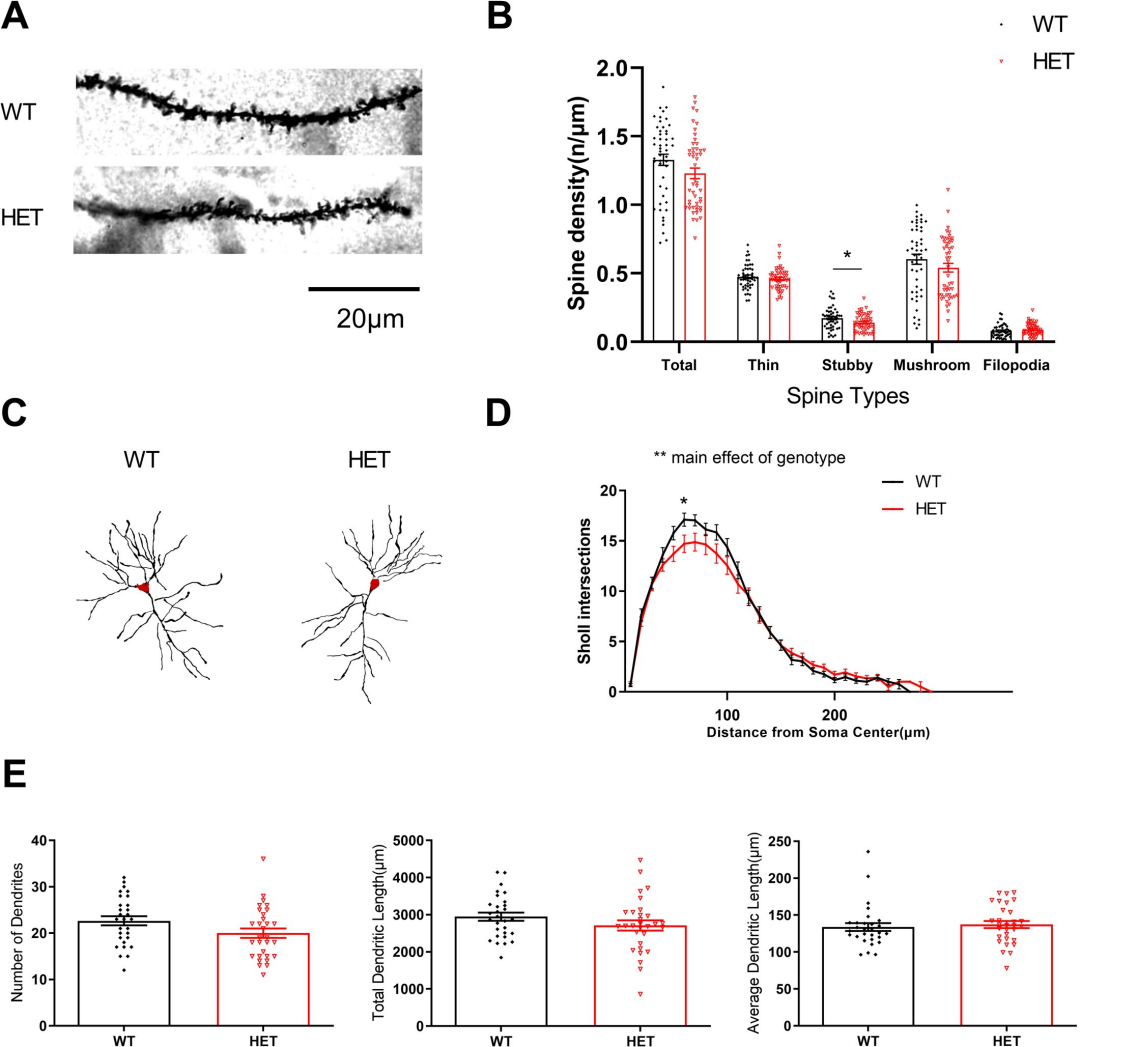
Sholl analysis and spine density analysis in mouse hippocampal neurons. A) Representative images of WT and *Cul3* HET global knockout hippocampal neuronal apical dendritic spines. Scale bar, 20 μm. B) Spine density analysis of WT and *Cul3* HET demonstrates no change of apical dendritic spine density except the stubby spine density decrease in *Cul3* HET mutant neurons (Total spine, F = 1.16, P = 0.0781; thin spine, F = 1.285, P = 0.3683; stubby spine, F = 1.641, P = 0.0267; mushroom spine, F = 1.319, P = 0.2034; filopodia spine, F = 1.015, P = 0.2525. WT N = 50/10 cells/mice; HET N = 50/10). *P<0.05, **P<0.01, ***P<0.001, unpaired student’s t-test; graphs depict mean ± SEM. C) Representative images of WT and *Cul3* HET global knockout mice hippocampal neuronal dendritic tree. Scale bar, 20 μm. D) Sholl analysis of WT and *Cul3* HET knockouts demonstrates decreased dendritic branching in *Cul3* HET mutant neurons, a main effect of genotype was observed (P = 0.002, WT N = 30/10 cells/mice; HET N = 30/10). *P<0.05, **P<0.01, ***P<0.001, 2-way ANOVA test; graphs depict mean ± SEM. E) Number of dendrites (left, F = 1.136, P = 0.0669), total dendritic length (middle, F = 1.62, P = 0.1832), and average dendritic length (right, F = 1.219, P = 0.6299) was unchanged in WT and *Cul3* HET global knockout hippocampal neurons (WT N = 30/10 cells/mice; HET N = 30/10). *P<0.05, **P<0.01, ***P<0.001, unpaired student’s t-test; graphs depict mean ± SEM.

*Cul3* heterozygous deletion did not significantly change total spine density, dendrite elaboration, or dendrite maintenance in hippocampal CA1 pyramidal neurons (Fig 5A and 5B). Spine density analysis of WT and *Cul3* HETs demonstrated no significant change of spine density except a slight stubby spine density decrease in *Cul3* HET mutant neurons (Fig 5A and 5B). Heterozygous loss of *Cul3* slightly decreased dendritic complexity as measured by the number of Sholl intersections at various distances from the soma (Fig 5C and 5D). No significant differences were observed in number of dendrites (Fig 5E, left), total dendritic length (Fig 5E, middle), or average dendritic length (Fig 5E, right). These data suggest that *Cul3* heterozygous deletion s only minimally alters dendrite complexity *in vivo*.

### *Cul3* heterozygous deletion significantly increases mEPSC frequency but not other electrophysiological measures within hippocampus

**Fig 6:**
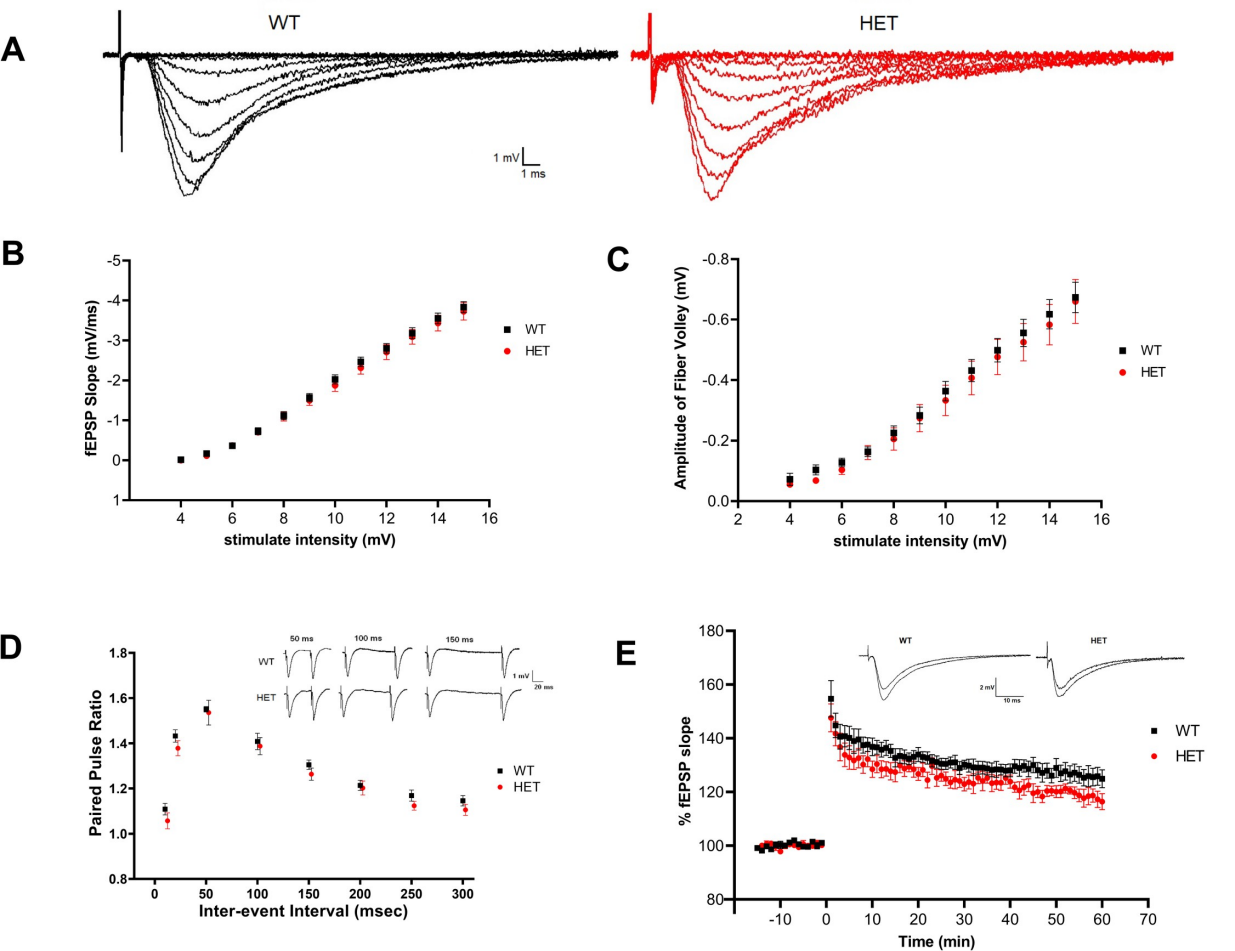
Insufficiency of *Cul3* does not affect baseline synaptic transmission, the presynaptic transmission, or LTP in hippocampus. A) Representative traces of field recording fEPSPs in hippocampal Schaffer-collateral pathway from *Cul3* HETs and WT littermates. B) Input output curves of evoked fEPSP slope versus stimulus intensity demonstrated no significant difference (P = 0.6141) in baseline evoked synaptic transmission between WT and HETs. C) No significant differences (P = 0.6434) were found in relationship between stimulus amplitude and fiber volley. D) Paired-pulse facilitation across different interstimulus intervals revealed no effect of genotype (P = 0.2816). E) Induction of LTP using high-frequency stimulation (3 x tbs) revealed no significant differences between WT and HETs (P = 0.0913). (WT/HET = 8 mice/ group, 3–6 slices/ mouse). *P<0.05, **P<0.01, ***P<0.001, 2-way ANOVA with repeated measures; graphs depict mean ± SEM.

Because hippocampus is an area of the brain implicated in social interaction and learning and memory [46, 47], we performed field recordings on dorsal hippocampal slices from 6–7 week old mice. Field recording data (n = 8 mice/group, 3–6 slices/mouse) suggested that insufficiency of *Cul3* does not affect the processes involved in baseline synaptic transmission, presynaptic transmission, or induction of LTP (Fig 6). No significant differences were found in input/output curves of evoked fEPSP slope versus stimulus intensity (Fig 6A and 6B), and analysis of fiber volley reflects that about the same number of axons were activated to give rise to a synaptic response (Fig 6C). No significant differences were found in the paired-pulse ratio (Fig 6D) or long-term potentiation (Fig 6E) between WT and HETs.

**Fig 7:**
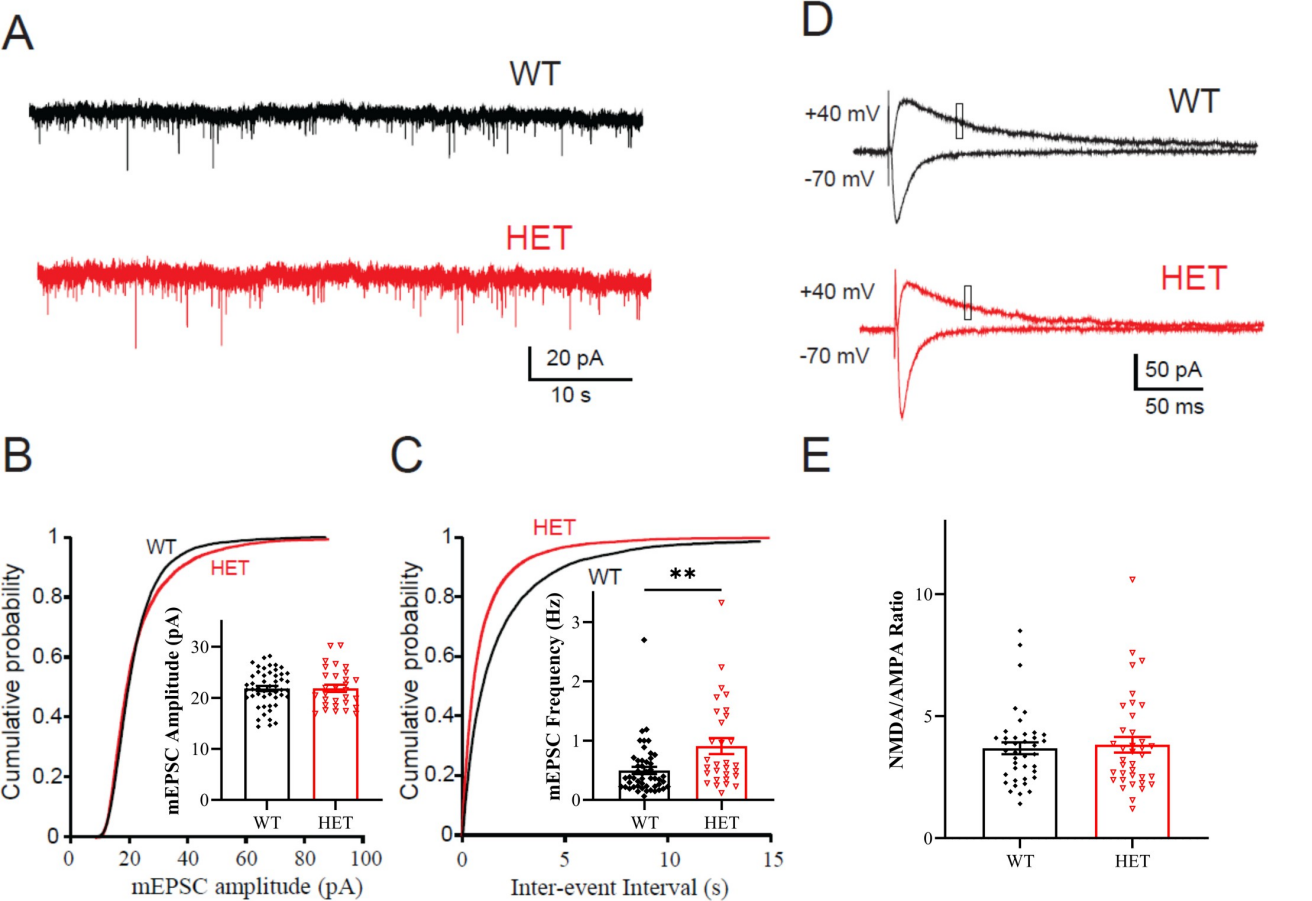
*Cul3* heterozygous deletion leads to increased mEPSC frequency in CA1 pyramidal neurons and no change in NMDA/AMPA ratio in mPFC. A) Representative traces of mEPSCs in dorsal hippocampal CA1 neurons from *Cul3* HETs and WT littermates. (WT N = 50/14 cells/mice; HET N = 30/11 cells/mice). B) Cumulative probability and summarized bar graph (inset) showing that the amplitude of mEPSC is comparable between genotypes (F = 1.169, P = 0.968). C) Cumulative probability and summarized bar graphs (inset) showing that the frequency of mEPSC is significantly increased in *Cul3* HET neurons compared with WT neurons (F = 2.978, P = 0.0023). D) Representative traces of eEPSCs recorded in mPFC layer 5 neurons following stimulus in layer 2/3. AMPAR current was measured as the peak response when the cells were held at -70 mV; NMDAR current was measured as the mean current over a 5-ms window, 50 ms after stimulation in L2/3. (WT N = 40/6 cells/mice; HET N = 37/6 cells/mice). E) Summarized bar graphs showing that NMDA/AMPA current ratio is comparable between genotypes (F = 1.624, P = 0.7178). *P<0.05, **P<0.01, ***P<0.001, unpaired student’s t-test; graphs depict mean ± SEM.

Data analysis of mEPSCs recorded from hippocampal CA1 neurons indicates that the amplitude of mEPSC was comparable between genotypes (Fig 7A and 7B), but the mEPSC frequency was significantly increased in heterozygous cells compared with that in WT cells (Fig 7A and 7C).

### *Cul3* heterozygous deletion does not significantly alter NMDA/AMPA ratio within mPFC

Because an initial report suggested a decrease in NMDAR-mediated input/output curves with unchanged AMPAR-mediated EPSC input/output curves in layer 5 prefrontal cortex pyramidal neurons with conditional heterozygous *Cul3* deletion [21] and its implication in autism and learning/memory [48, 49], we examined stimulus-evoked NMDA/AMPA EPSC ratio in layer 5 PFC pyramidal neurons. Using NMDA/AMPA ratio allows for internal normalization of stimulus intensity using AMPAR responses in the same neuron; input/output curves, on the other hand, do not control for variations in stimulus electrode resistance that can vary from slice to slice and from experiment to experiment [50–52]. *Cul3* heterozygous deletion does not significantly change basic membrane properties in mPFC neurons including resting membrane potential, total capacitance, or input resistance (p > 0.05 for all values. See Table 1). The NMDA/AMPA current ratio was recorded in layer 5 (L5) mPFC pyramidal neurons by stimulating in layer 2/3 (L2/3). The NMDA/AMPA current ratio was comparable between WT and *Cul3* HET neurons (Fig 7D and 7E).

**Table 1:** Basic membrane properties of mPFC neurons.

	RMP (mV)	Cm (pF)	Rm (MΩ)
WT	-60.3 ± 0.9 (38)	155 ± 7 (40)	130 ± 9 (40)
HET	-60.7 ± 1.0 (34)	157 ± 8 (37)	129 ± 9 (37)

Abbreviations: RMP, resting membrane potential; Cm, membrane capacitance; Rm, membrane resistance.

### *Cul3* heterozygous deletion leads to alterations in protein expression

**Fig 8:**
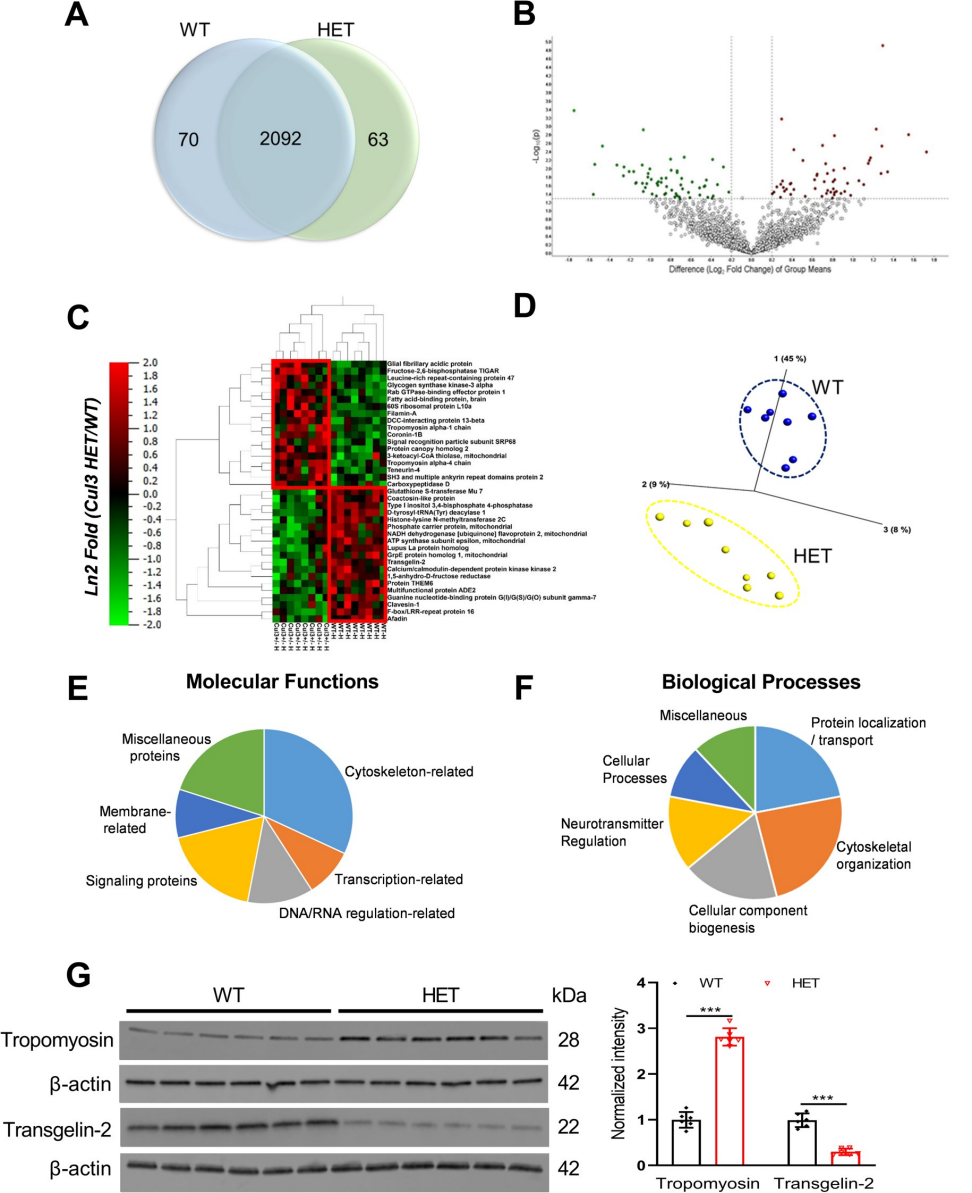
*Cul3* heterozygous deletion in mouse hippocampus induces differential expression of proteins. A) Global protein changes in the hippocampus of adult (8–10 weeks old) WT vs *Cul3* HET mice as represented by Venn diagram. 133 (63↑70↓) proteins were found to be statistically changed in the hippocampus of the *Cul3* HET vs WT out of the total of 2,092 proteins. The Venn diagram depicts the total number of proteins identified across both groups, in addition to those proteins found to be significantly changed as a result of *Cul3* HET deletion. B). Volcano plot demonstrating the distribution of the entire data set of proteins with upper limits indicating statistically significant changes depicted in red and green points. C) 2-dimensional heat map of top 37 proteins demonstrates the proteins which are increased (red) or decreased (blue). D) Principle Component Analysis (PCA) demonstrating a tight clustering of the samples across each group. E & F) Gene ontology (GO)-annotated molecular function (E) and biological processes (F) were identified for differentially expressed proteins between the WT and *Cul3* HET. System analysis used the top 133 statistically significant proteins, and the resultant pie charts are indicative of the normalized percentage of proteins associated with each category within molecular functions and biological processes. G) Representative western blots (left) and pooled densitometry quantification data (right) for Tropomyosin and Transgelin-2 in hippocampal brain regions (F = 1.209, P<0.0001 for Tropomyosin, F = 4.38, P<0.0001 for Transgelin-2. Male WT = 3, HET = 3; female, WT = 3, HET = 3). *P<0.05, **P<0.01, ***P<0.001, unpaired student’s t-test; graphs depict mean ± SEM.

Global proteomics workflow was carried out on the hippocampus utilizing the 1D-PAGE-LC-MS2 approach. Proteins extracted from the hippocampi of each animal were loaded onto a 1-dimensional denaturing polyacrylamide gel. The resulting lane for each individual sample was cut into 6 equally sized MW fractions and digested with trypsin, and the resultant peptides from each fraction were separated using nano-HPLC and analyzed using a high resolution MS2 mass spectrometer [36]. Following this approach, we identified 2,092 proteins with >99% confidence and <1% FDR. Quantification and pairwise statistical analysis yielded 133 differentially abundant proteins, with 63 increased and 70 decreased in *Cul3* HETs. A Venn diagram depicts the total number of proteins identified across both groups, while also highlighting the significantly changed proteins (Fig 8A). A volcano plot (Fig 8B) demonstrates the distribution of all data points, with upper limits (above the line) showing statistically significant changes and outer limits (to the right and left of each vertical line) showing significant fold changes. Fold change is visualized as log-fold, with a cut-off value ±1.5 applied to fold changes. To visualize the most significant changes in the proteome, we have included a q-value (false discovery rate adjusted p-value) as an added filter to highlight in the multi-dimensional plots for a cleaner qualitative view of the higher confidence data, narrowing to 37 differentially abundant proteins. Qualitative analyses were carried out on the highly significant 37 proteins, a 2-dimensional hierarchical clustering analysis heat map and a principal component analysis (Fig 8C and 8D). Both of these analyses verify the statistical significance of the differences recognized in proteins both between the two groups, as well as for each protein across the different animals. As can be appreciated, there is a close clustering of proteins of the two different groups (Fig 8D).

The top 133 differentially abundant proteins were next subjected to a systems biology analysis. Systems analysis allowed us to identify the key gene ontology (GO) annotations, thus focusing on the molecular functions and biological processes associated with the loss of *Cul3* allele in the *Cul3* HETs. The pie charts were generated to visualize the more common molecular functions and biological processes in which the differentially expressed proteins tended to occur (Fig 8E). A molecular functions analysis highlighted that most of the differentially expressed proteins were known to associate with several key functions such as cytoskeleton-related, signaling, membrane transport-related, transcription-related, synaptic transmission, and also miscellaneous functions. Similarly, biological processes associated with these proteins indicate that several primary cellular changes are occurring, including cytoskeletal organization, protein localization/transport, and neurotransmitter regulation (Fig 8F). Next, we confirmed proteomic findings via western blot analysis carried out on cytoskeletal associated proteins associated with actin stabilization such as Tropomyosin and Transgelin-2. Western blot confirmed significantly higher protein levels of Tropomyosin and decreased Transgelin-2 in the *Cul3* HETs (Figs 8G and S3).

In contrast to our original hypothesis, we did not see a change in RhoA protein expression levels in the *Cul3* heterozygous mutant mice compared to WT in either our proteomic experiment or our more direct immunoblot experiment.

## Discussion

Loss of function mutations in *CUL3* are thought to be an important risk factors for ASD patients [2, 3, 53–56]. We developed *Cul3* global knockout mice to determine the role of *Cul3* heterozygous loss-of-function in mammalian brain; unfortunately the complete knockout of *Cul3* resulted in lethality as previously described [20, 45], thus we focused on studying the effect of *Cul3* heterozygous knockout on the morphological, functional and behavioral alterations resulting from insufficiency of CUL3.

Overall, our results indicate that *Cul3* heterozygous deletion does not affect general measures of growth, motor or sensory abilities, repetitive behavior, reciprocal social interaction, social target approach, or novel object recognition, though they do demonstrate significantly decreased spatial object recognition. *Cul3* heterozygous deletion slightly affects dendrite complexity/branching, but does not affect total dendrite length or spine density. Analyses of synaptic transmission demonstrated increased mEPSC frequency in area CA1 of hippocampus, no change in mEPSC amplitude, unchanged baseline synaptic transmission, and no change in paired pulse ratio. *Cul3* heterozygous deletion does not alter mPFC NMDA/AMPA ratio. *Cul3* heterozygous deletion leads to alterations in cytoskeleton-related protein expression, among others. Overall, our data reveal that *Cul3* heterozygous deletion does not cause major morphological changes, synaptic transmission changes, or behavioral abnormalities in the adult mice brain.

We have compared our results with the recent published studies which focused on *Cul3* genetic mouse models and summarized them into a table (S2 Table in S1 File). Our data indicate that body weight is only decreased in female *Cul3* HETs at a single timepoint, which is largely consistent with what was discovered in *Cul3* global HETs by Morandell et al. [24]. Amar et al. [23] found significantly decreased body weight in both male and female heterozygous adult mice with exon 6 insertion mutation via CRISPR. Rapanelli et al. did not find a significant change of body weight in conditional heterozygous *Cul3* knockout mice using an Emx1-Cre to reduce *Cul3* expression in cortex and hippocampus [21]. In our hands *Cul3* heterozygous deletion did not significantly influence the adult weight of mice in general. Differences among studies may be explained by the use of different mouse models and time-frames analyzed.

Locomotor activity measured over 2 h was unchanged in *Cul3* HETs vs. WT littermates, which is consistent with Dong et al. using GFAP-cre [22] and Amar et al. using exon 6 insertion HETs [23]. Locomotor activity was increased in Rapanelli et al. using Emx1-cre conditional HETs [21]. In our hands, no difference in total distance travelled or average velocity of movement was observed during 10 min in an open field arena between *Cul3* HETs and WT littermates, consistent with the results of Morandell et al. [24] and Dong et al. [22] over a 30-min time period, while *Cul3* HETs showed increased total distance and speed in open field from Amar et al. [23] and Rapanelli et al. [21]. In our global *Cul3* heterozygous deletion model, two different tests of locomotor activity found no significant differences.

Motor coordination on the accelerating rotarod was normal in our *Cul3* HETs compared to WT littermates, which was also unchanged in GFAP-Cre/*Cul3*^f/-^ mice from Dong et al. [22] and Emx1-Cre/*Cul3*^f/-^ mice from Rapanelli et al. [21]. Defects in motor performance and coordination were reported, however, in *Cul3*^*+/−*^ mice from Morandell et al. [24].

No difference in time to paw-lick response was observed on the hotplate sensory task between our *Cul3* HETs and WT littermates. Time spent in the mouse scent interaction zone was unchanged between *Cul3* HETs and WT littermates in the odor discrimination and habituation test, which was also unchanged in Dong et al. [22], both wildtype and *Cul3*^*+/−*^ animals successfully recognized newly and already presented odors in Morandell et al. [24]. Time spent and distance travelled in center zone of an open field arena is increased in *Cul3* HETs in our results, which is unchanged in Rapanelli et al. [21] , Amar et al. [23], and decreased in Dong et al. [22]. Our additional tests of anxiety-related behaviors including dark/light and elevated plus maze both showed no change, consistent with a conclusion of no change in anxiety-related behaviors. Thus, we interpret our 3 tests of the anxiety-related domain as showing no anxiety-related phenotype. Results of other studies are limited to one or two anxiety-related tasks and for the most part do not demonstrate consistent anxiety-related behavioral changes.

No change in startle response or Prepulse inhibition (PPI) was observed between *Cul3* HETs and WT littermates, which is consistent with Amar et al. [23]. Rapanelli et al. [21] found significantly decreased PPI at one of three stimulus intensities in Emx1-Cre/*Cul3*^f/-^ mice. Altogether, our results indicate *Cul3* heterozygous deletion does not generally alter the locomotor, sensory, anxiety-related, or olfactory ability in mice.

We did not see a significant change in time spent grooming in *Cul3* HETs compared to WT, findings consistent with most of the previous studies [21–23]. No difference in number of marbles buried was observed in our marble burying task. Thus, we conclude no difference in repetitive behaviors in *Cul3* HETs.

We did not find differences in time spent in direct physical contact during a reciprocal social interaction task examining sex-matched WT/WT and HET/HET pairs of mice in an open arena. We consider this direct, reciprocal social interaction task ethologically relevant. Time spent approaching a caged social target in an open arena is also comparable between *Cul3* HETs and WT littermates. Our data are similar to those of Amar et al. showing normal sociability (no difference in preference for a social target vs. an inanimate target) with decreased social recognition memory (decreased preference for a novel social target vs. a familiar social target) [23] in exon 6 insertion mutant *Cul3* HETs, and to those of Morandell *et al*. [24] showing normal preference for a social target versus a non-social/inanimate target (i.e. normal “sociability”) with decreased preference for a novel social target versus a familiar social target (i.e. altered social recognition memory) in *Cul3* global HETs. Our lack of observed social deficits on multiple tasks contrasts with Rapanelli *et al*. who showed decreased social approach, decreased social preference, decreased direct social interactions in their Emx1-cre conditional HET model [21]. Our findings also contrast with Dong et al. GFAP-Cre *Cul3* conditional heterozygous knockout mice that demonstrated differences in preference for a social target versus a non-social/inanimate target (i.e. “sociability”) as well as preference for a novel social target versus a familiar social target (i.e. social recognition memory) [22].

When examining the social behavior domain, it is critical to distinguish between preference for a social target versus an inanimate target (i.e. “sociability”) and preference for a novel social target versus a familiar social target (i.e. social recognition memory). In the 3-chamber test of sociability, a lack of preference for a social target versus an inanimate target should be interpreted as decreased “sociability” [57]. A lack of preference for a novel social target versus a familiar social target, however, should not interpreted as decreased “sociability” or decreased social interaction; instead this is more a test of social recognition learning and memory. So we discount interpretations of the 3-chamber sociability test that interpret social recognition memory as a deficit in social interaction or sociability. Morandell et al., for example, found no alteration of preference for a social target versus an inanimate target (i.e. “sociability”), consistent with our lack of difference in both reciprocal social interaction and in social approach of a caged target mouse. Morandell, et al., however, did see a difference in social recognition memory in global *Cul3* heterozygous mutant mice [24], a behavioral domain that we did not test in our study.

Sex was also included as an independent biological variable in all statistical analyses. We observed an expected significant main effect of sex in body weight. Although we discovered a main effect of sex in five behavioral tasks: open field, olfactory preference for social odors, startle response, reciprocal social interaction and cage conspecific test, these significant differences are largely between the WT male and WT female groups. When comparing WT males vs HET males, or WT females vs HET females group, we did not discover any significant difference (See S2 Fig and S1 Table in S1 File for details).Our study does not directly contradict findings in conditional cre-dependent deletion *Cul3* heterozygous models. This is because one cannot compare a partial, conditional deletion to a global deletion from conception. Conditional partial deletion can inform *Cul3* function in the cell populations or brain regions in which they are deleted; such models, however, lack construct validity in terms of timing, location, and completeness of deletion of *Cul3*. Many of the cre-driven conditional *Cul3* heterozygous mutants do exhibit behavioral and other phenotypes that differ from the global *Cul3* heterozygous mutants we tested. Again, such differences may be related to the use of cre driver lines in terms of timing, location, and completeness/mosaicism of the deletion. Additional potential explanations could be subtle differences in behavioral task protocols, housing conditions, handling conditions, order of testing, or other difficult to control effects.

Our data indicate that heterozygous deletion of *Cul3* does not cause obvious, major behavioral differences. We do, however, detect impaired spatial objection recognition in a single task. Altered spatial learning/memory may have some face validity for humans with Cul3 loss-of-function mutations because some of these patients also exhibit intellectual disability [56, 58–60]. The times spent with objects, following extensive habituation, in our spatial/novel object recognition task were seemingly low (∼5 s); yet our findings are clearly statistically significant and differ from WT mouse behavior. Several previous studies examining spatial object recognition memory report roughly similar times spent with habituated objects [16, 61–63]. Future studies are needed to confirm whether this finding is robust, reproducible, and consistent across different spatial learning and memory tasks. No obvious impairment of spatial working memory in GFAP-cre conditional *Cul3* HETs was observed in Dong et al. [22]. Spatial learning/memory deficits in global *Cul3* heterozygous mutants require further study.

Our neuronal morphology analysis revealed that *Cul3* heterozygous deletion causes a slight decreased dendritic complexity but does not drastically affect dendritic branching or spine density in hippocampal neurons, which is consistent with *Cul3* HET mice from Morandell et al. [24] and consistent with Emx1-Cre/*Cul3*^f/-^ mice in unaltered dendritic branching in PFC pyramidal neurons at 6 week age in Rapanelli study [21], though they do show decreased spine density in PFC neurons. Increased apical spine density and unchanged basal spine density were observed in GFAP-Cre/*Cul3*^f/-^ mice hippocampal CA1 pyramidal neurons at postnatal day 60 (P60) in Dong et al. [22]. These differences may be explained by the different mouse model, brain regions and time frame analyzed. We conclude that *Cul3* heterozygous deletion causes a slight decreased dendritic complexity but does not cause major neuronal morphology changes in area CA1 hippocampal pyramidal neurons in adult *Cul3* HET brains.

Our data demonstrate that *Cul3* heterozygous deletion increases mEPSC frequency but does not affect the amplitude recorded from hippocampal CA1 neurons, a finding confirmed by both GFAP-Cre/*Cul3*^f/-^ and NEX-Cre/Cul3^f/-^ mice hippocampal neurons [22].

Because Rapanelli et al. demonstrated a decrease in NMDAR-mediated evoked input/output curves in whole cell recordings in Layer 2/3 of mPFC, we examined NMDAR-mediated evoked synaptic transmission by measuring the NMDA/AMPA ratio. The NMDA/AMPA ratio in layer 5 pyramidal neurons evoked by layer 2/3 stimulation was not significantly altered in *Cul3* HET neurons in our study. Thus, we did not replicate a change in NMDAR-mediated evoked synaptic transmission in the mPFC shown by Rapanelli et al. [21] using an approach that normalizes stimulus intensity to the AMPA Receptor-mediated responses and is a more widely used approach to examine for changes in either NMDA or AMPA-Receptor-mediated synaptic changes.

Our extracellular field recording data from area CA1 of hippocampus indicate no change in baseline synaptic transmission (input/output curves) and no changes in paired-pulse ratio or long-term potentiation. Only Dong *et al*. showed a decrease in paired-pulse ratio in area CA1 of hippocampus in their GFAP-Cre *Cul3* conditional heterozygous knockout mice [22]. Our data support no changes in baseline transmission, paired-pulse ratio, or LTP. These findings and others make the interpretation of why we see increased mEPSC frequency in area CA1 of hippocampus difficult. Increased mEPSC frequency could mean an increase in functional excitatory synapses, yet we see no increase in spine density or dendritic length. It could also signify a change in presynaptic release probability, yet we see no change in paired pulse ratio, a measure that typically would change with changes in presynaptic release probability. Thus, the only explanation we can offer would be a change in the number of functional excitatory synapses that are not represented structurally as a change in spine density or dendritic length..

Global discovery-based proteomics studies were conducted to gain further insights into dysregulated proteins following *Cul3* heterozygous deletion. Our proteomic analysis identified 133 significantly differential proteins in the hippocampus upon heterozygous deletion of *Cul3* in mice. We included a q-value (false discovery rate adjusted p-value) between 0.05–0.10 as an additional filter, thus narrowing to 37 differentially expressed proteins. Our findings highlight several significant biological processes affected such as cytoskeletal organization, neurotransmitter regulation, cellular component biogenesis and protein localization/transport by Gene ontology analysis. Similarly, the significant molecular functions affected as a result of *Cul3* heterozygous deletion are related to cytoskeleton, synaptic transmission, transcription, and signaling. These findings are validated by western blot on two dysregulated cytoskeletal associated proteins, Tropomyosin [64] and Transgelin-2 [65]. Tropomyosin is an actin binding protein and plays an important role in regulating the structural diversity of the actin filaments. Tropomyosins have also been reported to play significant role in neuronal polarity, and neurite branching and extension [66]. Likewise, Transgelin-2 is an actin-binding protein that induces actin polymerization and stabilizes the actin cytoskeleton [67, 68]. Many studies suggest that autism and intellectual disabilities often involve defects in the regulation of actin cytoskeleton [69–74]. Further studies will be needed to begin to understand the implications of altered protein expression levels in the *Cul3* heterozygous mouse model.

Several studies have recently reported the dysregulation of cytoskeletal proteins as a result of loss of *Cul3* in their mouse model. Amar *et al*. also identified dysregulation of several cytoskeletal processes by *Cul3* heterozygous deletion [23] and reported three upregulated proteins (Vimentin, Plastin 3 and Inter-nexin). Although we did not detect Vimentin and Internexin, Plastin-2 (an isoform of Plastin-3) was found to be significantly increased in our dataset. Dong *et al* reports that hyperfunction of glutamatergic pathways are a patholological mechanism and showed upregulation of several SNARE proteins such as VAMP1, NSF and α/β-SNAP in their GFAP-Cre *Cul3* conditional heterozygous deletion model [22]. Though we detected VAMP1 and SNAP proteins in our proteomic dataset, they were not significantly altered in our *Cul3* heterozygous mice. Furthermore, eIF4G1 protein was detected in our dataset, however was not significantly different between the two groups [75]. Morandell *et al*. studied the developmental consequences of *Cul3* haploinsufficiency and also reported on dysregulation of actin and tubulin-associated proteins, Plastin-3 and MAP2 [24]. Rapanelli *et al*. also observed the dysregulation in cytoskeleton-related proteins, among others, in their Gene ontology analysis and reported on the upregulation of Smyd3, a histone methytransferase, contributing to the molecular alterations and behavior deficits in *Cul3*-deficient mice [21]. We did not detect Smyd3 in our proteomic dataset. As can be appreciated, proteomic analysis can be somewhat variable and can present several limitations including lack of confirmable detection of a large fraction of proteins and overlooking low abundant proteins due to its limit of detection. In addition, protein levels can fluctuate over time and state in tissues. The differences observed in our dataset may also be attributed to different *Cul3* mouse models/time points across different studies.

Overall, our proteomic results highlight the importance of cytoskeletal integrity and supports the notion that during *Cul3* haploinsufficiency multiple cytoskeletal proteins are impacted, consistent with the other published studies. Further studies are required to better define a role for the differentially expressed proteins in any phenotypes associated with the heterozygous deletion of *Cul3* in mice. In conclusion, our study demonstrates that *Cul3* heterozygous deletion impairs spatial object recognition memory, alters actin cytoskeleton-related and signaling proteins, and increases mEPSC frequency in hippocampus; it does not, however, cause major CA1 neuronal morphology, functional, or behavioral abnormalities in adult global *Cul3* heterozygous mice.

In contrast to our original hypothesis, we did not see a change in RhoA protein expression levels in the *Cul3* heterozygous mutant mice compared to WT. First, our proteomic analysis did not detect RhoA among the peptides analyzed; this is not definitive proof that RhoA levels are unchanged because proteomic experiments only detect a fraction of the proteins in any given sample [76, 77]. Using direct immunoblots, we found no change in RhoA expression in *Cul3* heterozygous mutant cortex or hippocampus (Fig 1B and 1C). One previously published study noted an increase in RhoA levels in a *Cul3* heterozygous deletion model [23], while another study found increased RhoA levels in a conditional, partial forebrain deletion model [21]. Other papers that performed proteomics also did not detect RhoA protein [22, 24]. Still other papers did not examine RhoA levels at all [22, 24].

Robust and replicable phenotypes are critical to the study of genetic models of autism; this manuscript offers data in a model with strong genetic construct validity to confirm some previous behavioral, functional, and proteomic findings while not fully reproducing other reported findings.

## Supporting information

S1 Fig Original uncropped and unadjusted western blot results from Fig 1B. (TIF)

S2 Fig Sex differences in behavioral tests. (TIF)

S3 Fig Original uncropped and unadjusted western blot results from Fig 8G. (TIF)

S1 File (DOCX)

S1 Raw images (PDF)
