# Supplementary figures and images for: Effects of heterozygous deletion of autism-related gene Cullin-3 in mice
Source: PLoS One. 2023 Jul 10;18(7):e0283299. doi: 10.1371/journal.pone.0283299 (PMC10332626; doi:10.1371/journal.pone.0283299)

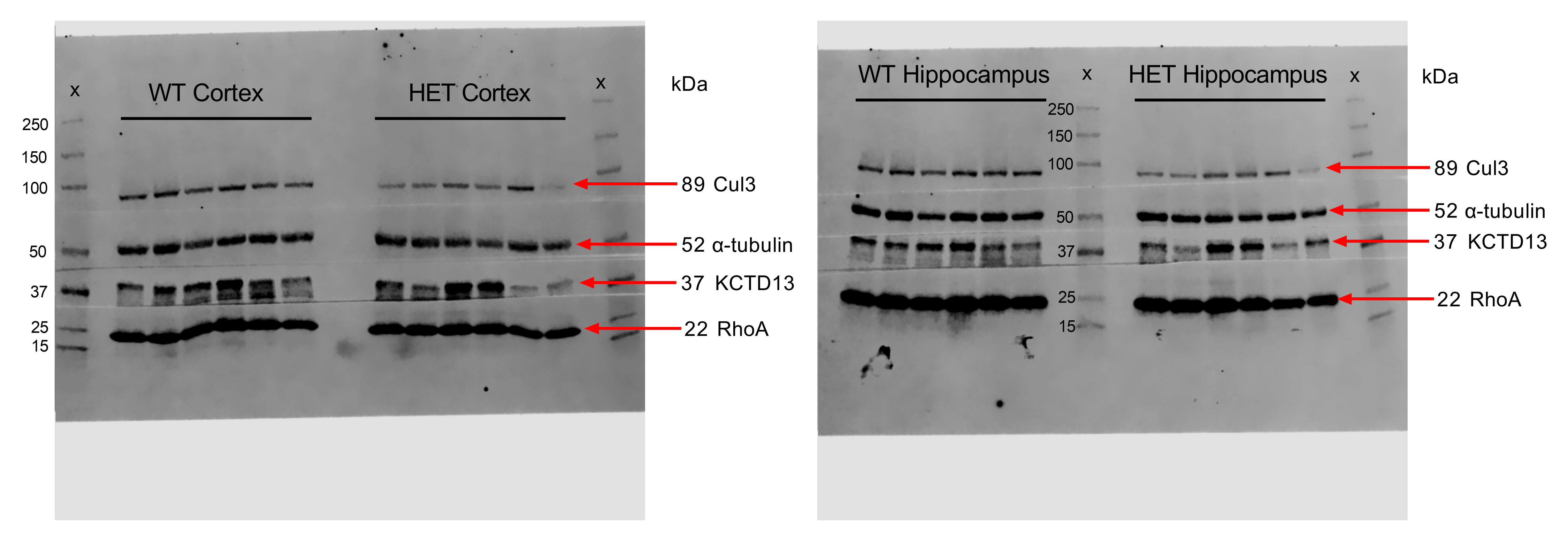

Supplement: S1 Fig — (TIF) [file pone.0283299.s001.tif]

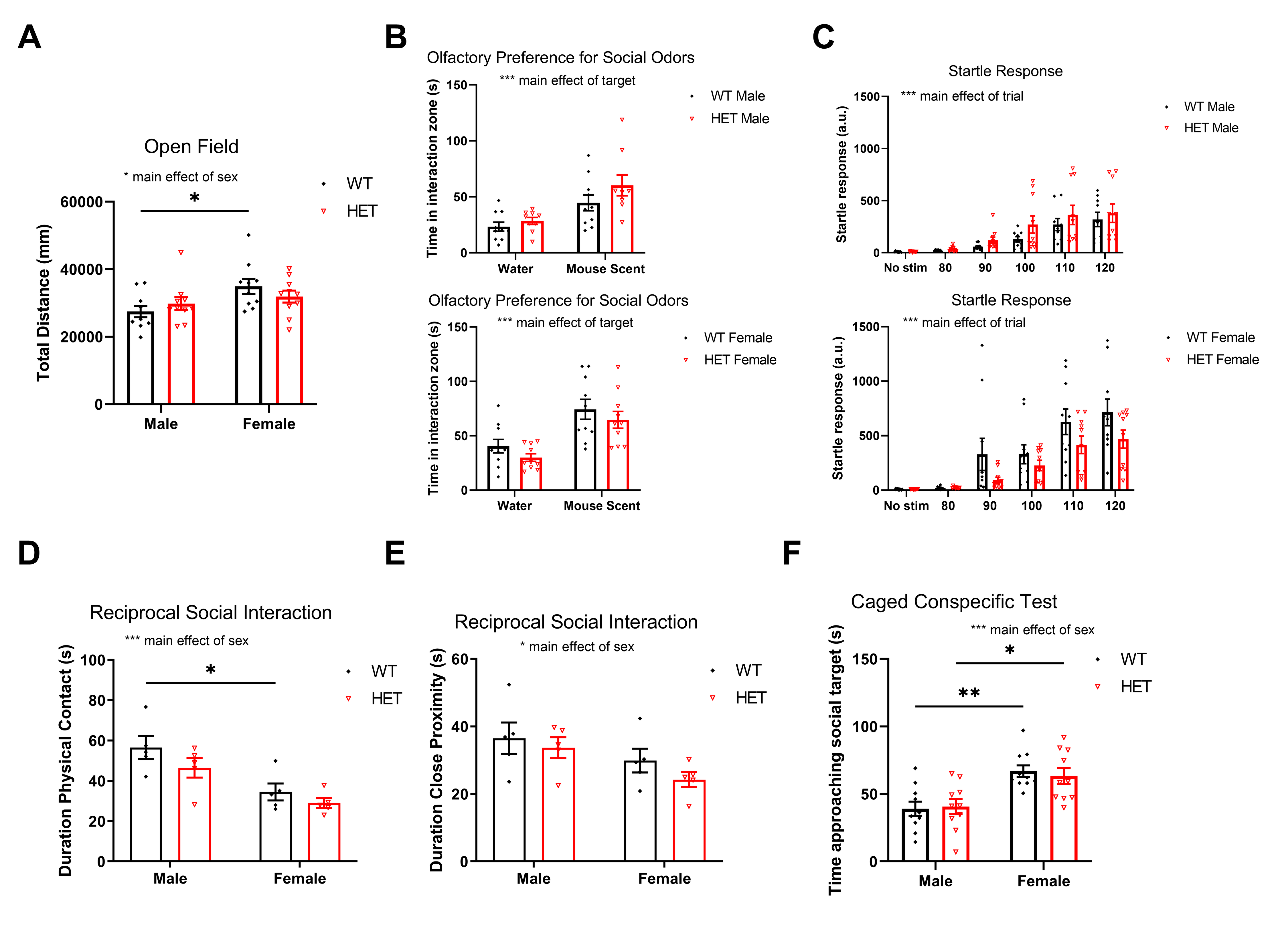

Supplement: S2 Fig — (TIF) [file pone.0283299.s002.tif]

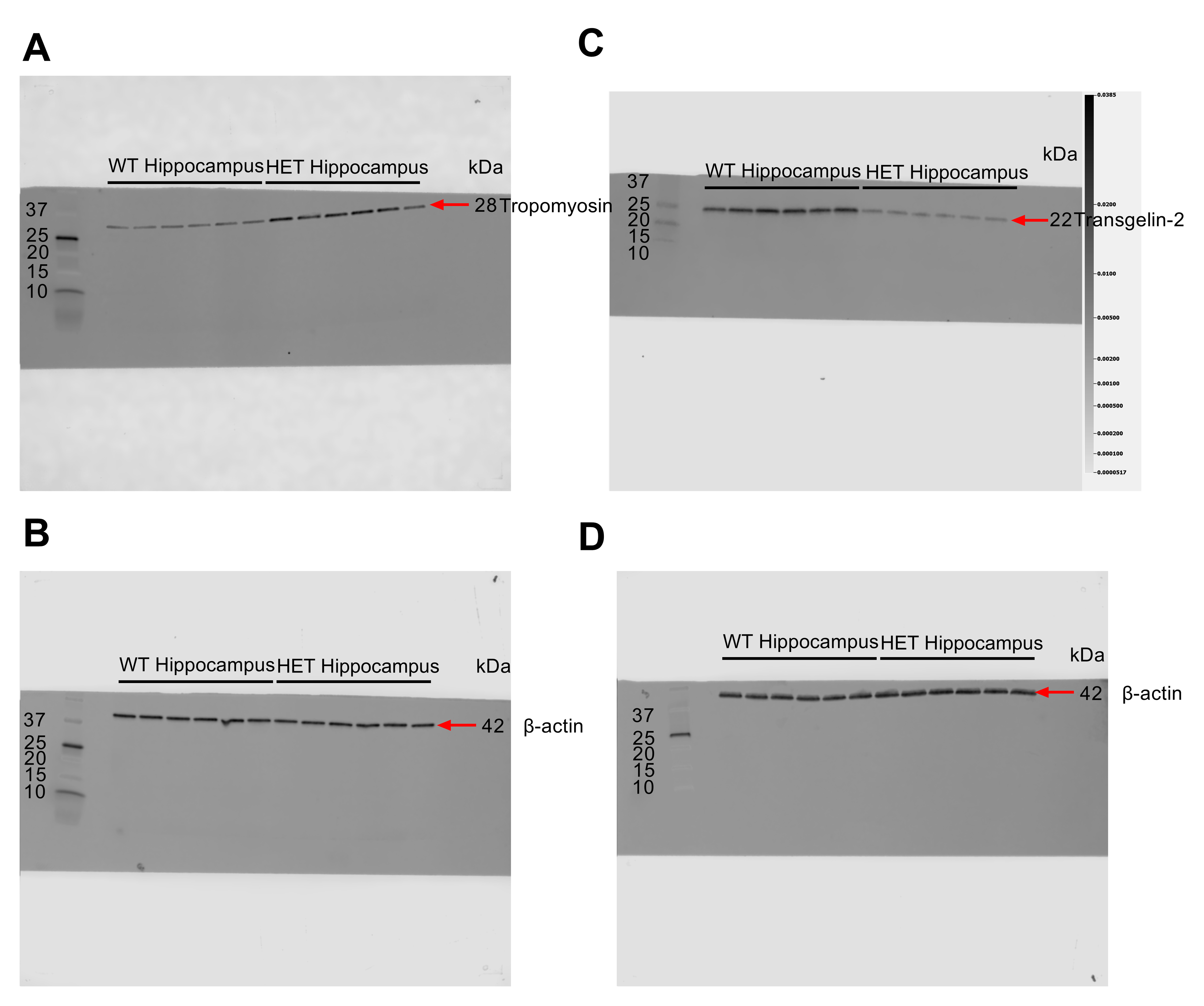

Supplement: S3 Fig — (TIF) [file pone.0283299.s003.tif]

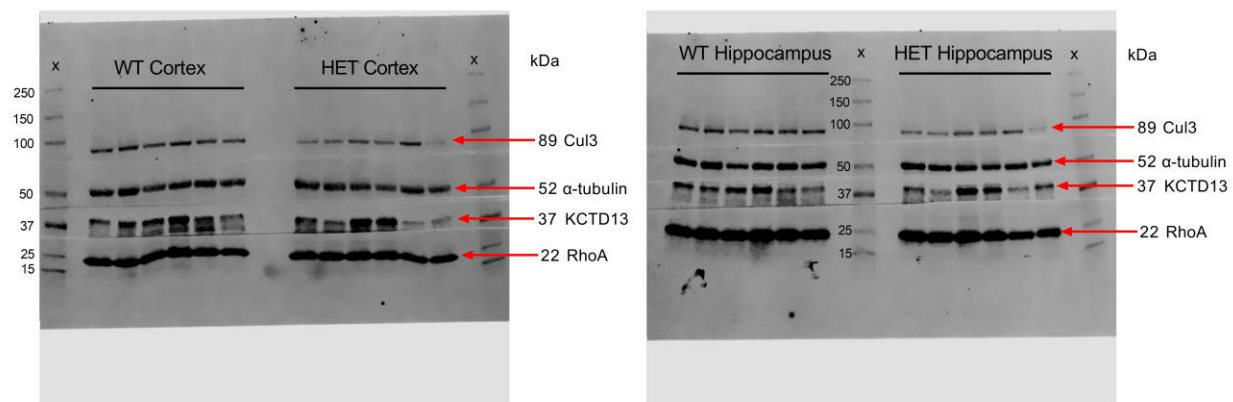

Figure S1.

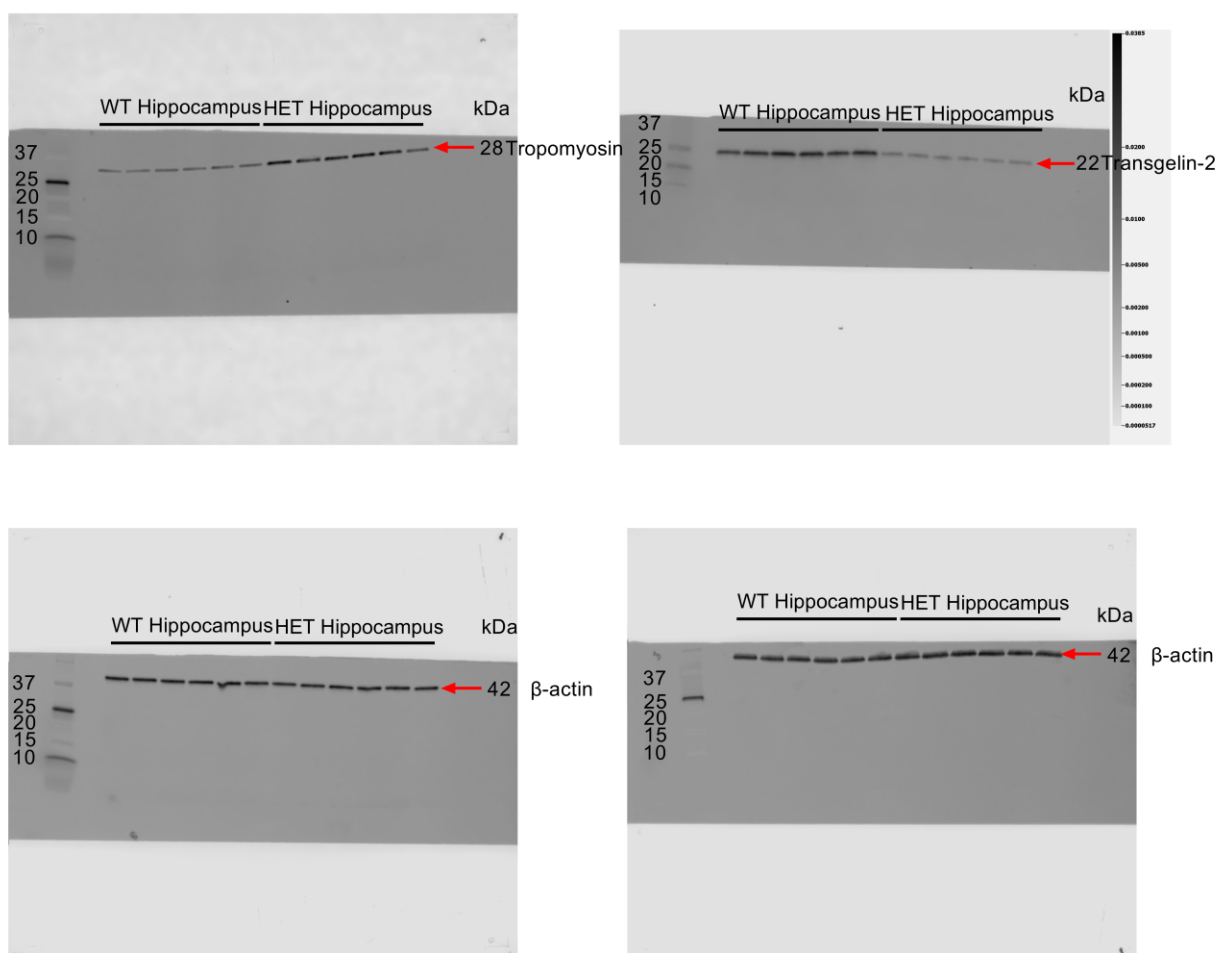

Figure S3.

Supplement: S1 Raw images — (PDF) [file pone.0283299.s005.pdf]
