# Supplementary material for: Effects of heterozygous deletion of autism-related gene Cullin-3 in mice
Source: PLoS One. 2023 Jul 10;18(7):e0283299. doi: 10.1371/journal.pone.0283299 (PMC10332626; doi:10.1371/journal.pone.0283299)
Supplement: S1 File — (DOCX) [file pone.0283299.s004.docx]

Supplementary material

**Effects of Heterozygous Deletion of Autism-related Gene *Cullin-3* in Mice**

Qiang-qiang Xia^1^, Angela K. Walker^2^, Chenghui Song^1^, Jing Wang^1^, Anju Singh^1^, James A. Mobley^3^, Zhong X. Xuan^1^, Jeffrey D. Singer^4^, Craig M. Powell^1*^

^1^Department of Neurobiology, University of Alabama at Birmingham Marnix E. Heersink School of Medicine, & Civitan International Research Center, Birmingham, AL, United States.

^2^Department of Neurology, University of Texas Southwestern Medical Center, Dallas, TX, United States.

^3^Department of Anesthesiology and Perioperative Medicine, University of Alabama at Birmingham Mass Spectrometry & Proteomics Shared Facility, Birmingham, AL, United States.

^4^Department of Biology, Portland State University, Portland, OR, United States.

*Corresponding author: Craig M. Powell ([craigpow@uab.edu](mailto:craigpow@uab.edu)).


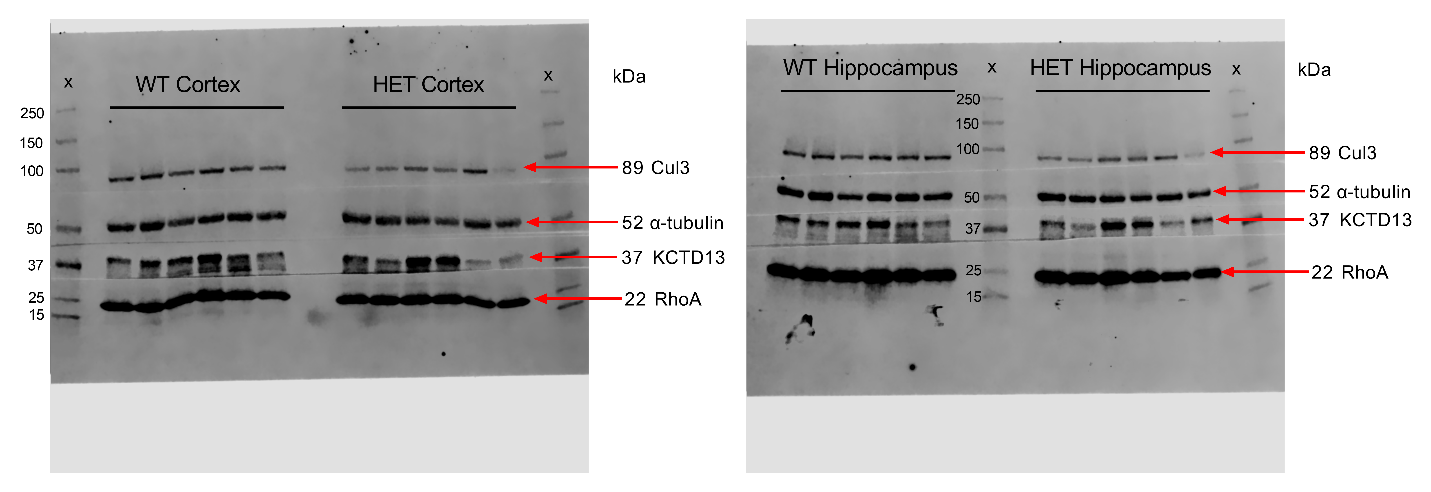


Figure S1. Original uncropped and unadjusted western blot results from Figure 1B. All the Cul3, α-tubulin, KCTD13 and RhoA bands of cortex or hippocampus samples are from same membrane.


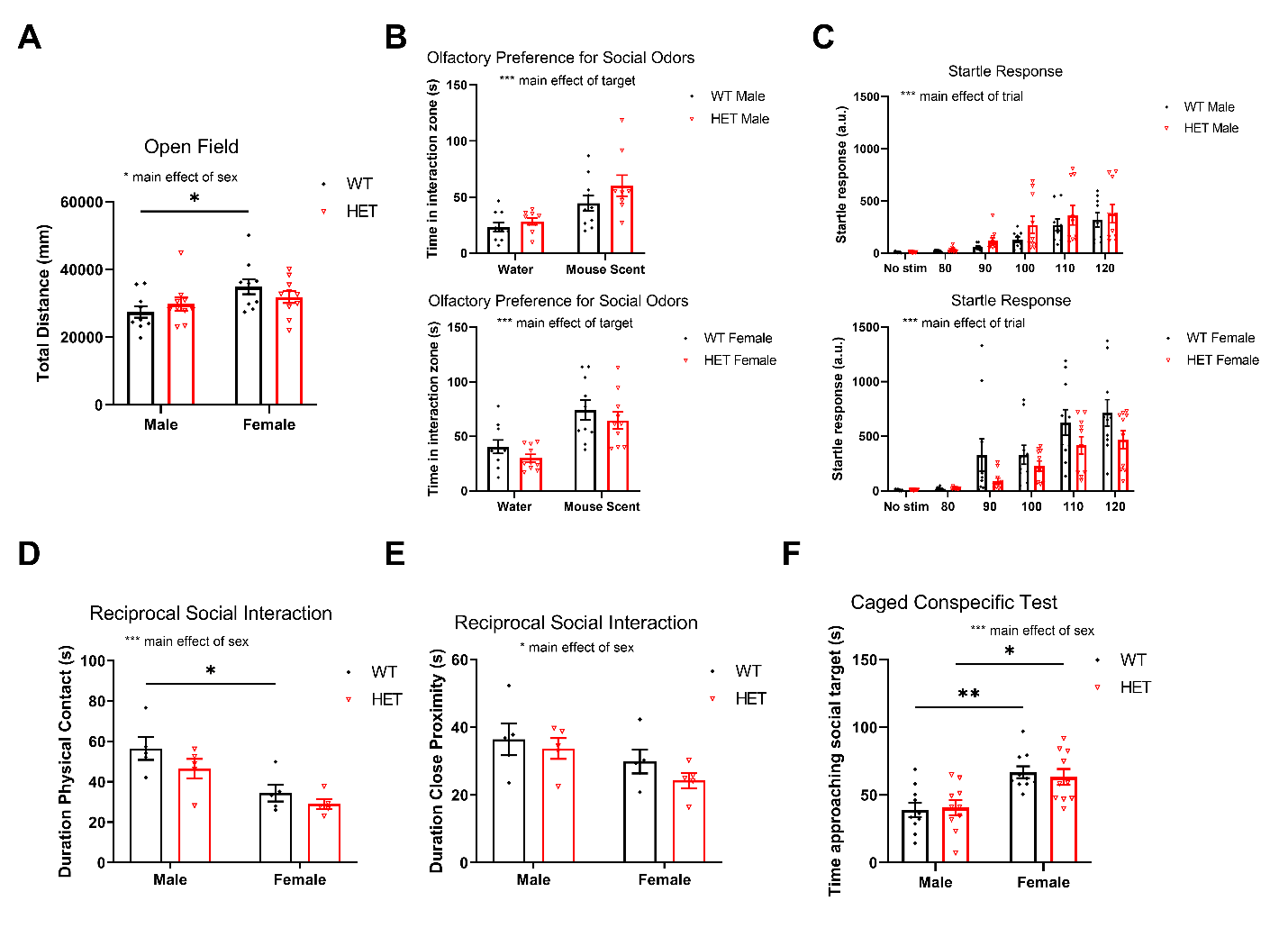


Figure S2. Sex differences in some behavioral tests were largely driven by WT group differences. A) Sex difference in total distance travelled in an open field arena. A main effect of sex was discovered (P=0.0167). WT male vs HET male, P=0.8207; WT male vs WT female, P=0.0413; WT female vs HET female, P=0.6718; HET male vs HET female, P=0.8658; male, WT=10, HET=10; female, WT=10, HET=10. B) No significant sex difference in time spent in the mouse scent interaction zone. A main effect of sex was discovered (P<0.0001). WT male vs HET male, P>0.9999; WT male vs WT female, P=0.0045; WT female vs HET female, P=0.9979; HET male vs HET female, P=0.0292; male, WT=10, HET=9; female, WT=10, HET=10. C) No significant sex difference in startle response in arbitrary units in response to white noise auditory stimulus pulse of various volumes in decibels (dB). A main effect of sex was discovered (P=0.0002). No stim. WT male vs No stim. HET male, P>0.9999; 80 WT male vs 80 HET male, P>0.9999; 90 WT male vs 90 HET male, P>0.9999; 100 WT male vs 100 HET male, P=0.999; 110 WT male vs 110 HET male, P>0.9999; 120 WT male vs 120 HET male, P>0.9999; No stim. WT female vs No stim. HET female, P>0.9999; 80 WT female vs 80 HET female, P>0.9999; 90 WT female vs 90 HET female, P=0.6796; 100 WT female vs 100 HET female, P>0.9999; 110 WT female vs 110 HET female, P=0.8571; 120 WT female vs 120 HET female, P=0.6156; male, WT=9, HET=10; female, WT=10, HET=10. D) No sex difference in time spent in direct physical contact during a reciprocal social interaction task examining sex-matched WT/WT pairs and HET/HET pairs of mice in an open arena. A main effect of sex was discovered (P=0.0004). (WT male vs HET male, P=0.5619; WT male vs WT female, P=0.0175; WT female vs HET female, P=0.9516; HET male vs HET female, P=0.0789; male, WT=5 pairs, HET=5 pairs; female, WT=5 pairs, HET=5 pairs). E) No sex difference in time spent in close proximity during a reciprocal social interaction task of genotype and sex-matched WT/WT and HET/HET pairs. A main effect of sex was discovered (P=0.0356). (WT male vs HET male, P=0.9949; WT male vs WT female, P=0.7408; WT female vs HET female, P=0.8505; HET male vs HET female, P=0.3688; male, WT=5 pairs, HET=5 pairs; female, WT=5 pairs, HET=5 pairs). F) No sex difference in time spent in proximity (approaching) a caged social target in an open arena. A main effect of sex was discovered (P<0.0001). (WT male vs HET male, P>0.9999; WT male vs WT female, P=0.0045; WT female vs HET female, P=0.9979; HET male vs HET female, P=0.0292; male, WT=10, HET=10; female, WT=10, HET=10). *P<0.05, **P<0.01, ***P<0.001, 2-way ANOVA or 3-way ANOVA test, graphs depict mean ± SEM. (See Supplementary table 1 for detailed statistics.)


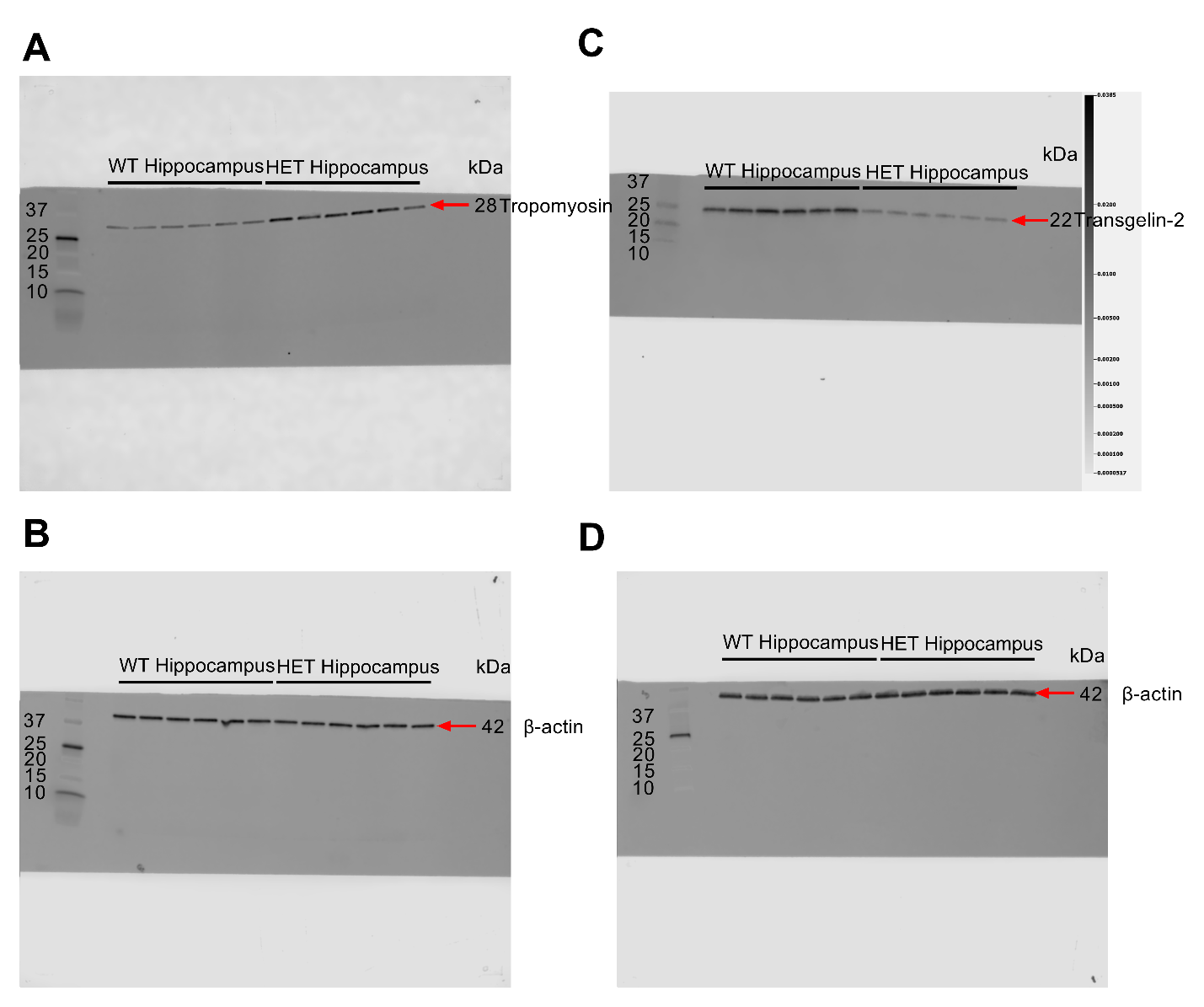


Figure S3. Original uncropped and unadjusted western blot results from Figure 8G. Panel A and B are from same membrane, panel C and D are from same membrane, although both pairs of them are captured at different time due to different hosts of antibodies, it did not affect the accuracy of comparison.

Supplementary table 1. Statistical analysis of body weight and behavioral studies.

| Parameter | Comparison | Results |
| --- | --- | --- |
| Body weight | Genotype, sex and age | 3-way ANONA, main effect of genotype: F(1,145)=23.14, P<0.0001; main effect of sex: F(1,145)=500.8, P<0.0001; main effect of age: F(4,145)=21.81, P<0.0001; genotype × sex interaction: F(1,145)=0.7025, P=0.4033; genotype × age interaction: F(4,145)=0.3063, P=0.8734; sex × age interaction: F(4,145)=3.644, P=0.0074; genotype × sex × age interaction: F(4,145)=1.723, P=0.148 |
| Locomotor habituation | Genotype, sex and bin | 3-way rmANONA, main effect of genotype: F(1,36)=0.0372, P=0.8482; main effect of sex: F(1,36)=1.33, P=0.2565; main effect of bin: F(23,828)=140.6, P<0.0001; genotype × sex interaction: F(1,36)=0.6271, P=0.4336; genotype × bin interaction: F(23,828)=0.9594, P=0.5171; sex × bin interaction: F(23,828)=0.985, P=0.4824; genotype × sex × bin interaction: F(23,828)=0.7925, P=0.7434 |
| Open field |  |  |
| Total distance | Genotype and sex | 2-way ANONA, main effect of genotype: F(1,36)=0.0349, P=0.8529; main effect of sex: F(1,36)=6.3, P=0.0167; genotype × sex interaction: F(1,36)=2.004, P=0.1655 |
| Time in center | Genotype and sex | 2-way ANONA, main effect of genotype: F(1,36)=5.156, P=0.0292; main effect of sex: F(1,36)=0.296, P=0.5898; genotype × sex interaction: F(1,36)=0.0651, P=0.8 |
| Distance in center | Genotype and sex | 2-way ANONA, main effect of genotype: F(1,36)=4.513, P=0.0406; main effect of sex: F(1,36)=1.749, P=0.1944; genotype × sex interaction: F(1,36)=0.9684, P=0.3316 |
| Time in periphery | Genotype and sex | 2-way ANONA, main effect of genotype: F(1,36)=3.834, P=0.058; main effect of sex: F(1,36)=0.8636, P=0.3589; genotype × sex interaction: F(1,36)=0.004, P=0.9499 |
| Rotarod | Genotype, sex and trial | 3-way ANONA, main effect of genotype: F(1,280)=1.963, P=0.1623; main effect of sex: F(1,280)=0.7946, P=0.3735; main effect of trial: F(7,280)=10.53, P<0.0001; genotype × sex interaction: F(1,280)=0.0922, P=0.7616; genotype × trial interaction: F(7,280)=1.678, P=0.1142; sex × trial interaction: F(7,280)=0.3539, P=0.928; genotype × sex × trial interaction: F(7,280)=0.3011, P=0.953 |
| Hotplate sensitivity | Genotype and sex | 2-way ANONA, main effect of genotype: F(1,35)=1.454, P=0.236; main effect of sex: F(1,35)=2.816, P=0.1022; genotype × sex interaction: F(1,35)=0.2066, P=0.6523 |
| Olfactory preference for social odors | Genotype, sex and target | 3-way ANONA, main effect of genotype: F(1,70)=0.001, P=0.9659; main effect of sex: F(1,70)=7.898, P=0.0064; main effect of target: F(1,70)=41.5, P<0.0001; genotype × sex interaction: F(1,70)=4.708, P=0.0334; genotype × target interaction: F(1,70)=0.3612, P=0.5498; sex × target interaction: F(1,70)=0.6718, P=0.4152; genotype × sex × target interaction: F(1,70)=0.262, P=0.6103 |
| Elevated plus maze | Genotype and sex | 2-way ANONA, main effect of genotype: F(1,36)=0.7412, P=0.395; main effect of sex: F(1,36)=0.0004, P=0.9847; genotype × sex interaction: F(1,36)=0.177, P=0.6765 |
| Dark/light box | Genotype and sex | 2-way ANONA, main effect of genotype: F(1,36)=0.019, P=0.8908; main effect of sex: F(1,36)=0.805, P=0.3756; genotype × sex interaction: F(1,36)=0.1186, P=0.7325 |
| Startle response | Genotype, sex and trial | 3-way ANONA, main effect of genotype: F(1,210)=1.604, P=0.2067; main effect of sex: F(1,210)=14.66, P=0.0002; main effect of trial: F(5,210)=32.82, P<0.0001; genotype × sex interaction: F(1,210)=12.04, P=0.0006; genotype × trial interaction: F(5,210)=0.5541, P=0.7351; sex × trial interaction: F(5,210)=2.293, P=0.0468; genotype × sex × trial interaction: F(5,210)=1.183, P=0.3188 |
| Prepulse inhibition | Genotype, sex and trial | 3-way rmANONA, main effect of genotype: F(1,36)=1.656, P=0.2063; main effect of sex: F(1,36)=0.1591, P=0.6923; main effect of trial: F(2,72)=162, P<0.0001; genotype × sex interaction: F(1,36)=3.36, P=0.0751; genotype × trial interaction: F(2,72)=4.002, P=0.0225; sex × trial interaction: F(2,72)=3.124, P=0.05; genotype × sex × trial interaction: F(2,72)=0.0518, P=0.95 |
| Grooming | Genotype and sex | 2-way ANONA, main effect of genotype: F(1,36)=0.047, P=0.8296; main effect of sex: F(1,36)=0.052, P=0.8211; genotype × sex interaction: F(1,36)=3.188, P=0.0826 |
| Marble burying | Genotype and sex | 2-way ANONA, main effect of genotype: F(1,36)=2.049, P=0.161; main effect of sex: F(1,36)=1.505, P=0.2279; genotype × sex interaction: F(1,36)=0.785, P=0.3815 |
| Reciprocal social interaction |  |  |
| Duration physical contact | Genotype and sex | 2-way ANONA, main effect of genotype: F(1,16)=3.062, P=0.0993; main effect of sex: F(1,16)=19.69, P=0.0004; genotype × sex interaction: F(1,16)=0.2674, P=0.6122 |
| Duration close proximity | Genotype and sex | 2-way ANONA, main effect of genotype: F(1,16)=1.44, P=0.2477; main effect of sex: F(1,16)=5.265, P=0.0356; genotype × sex interaction: F(1,16)=0.1688, P=0.6867 |
| Caged conspecific test | Genotype and sex | 2-way ANONA, main effect of genotype: F(1,36)=0.029, P=0.8651; main effect of sex: F(1,36)=22.33, P<0.0001; genotype × sex interaction: F(1,36)=0.2389, P=0.6279 |
| Novel and spatial object recognition |  |  |
| Baseline | Genotype, sex and object | 3-way ANONA, main effect of genotype: F(1,102)=1.993, P=0.1611; main effect of sex: F(1,108)=1.275, P=0.2615; main effect of object: F(2,102)=2.124, P=0.1248; genotype × sex interaction: F(1,102)=7.152, P=0.0087; genotype × object interaction: F(2,102)=0.198, P=0.8207; sex × object interaction: F(2,102)=0.9167, P=0.4031; genotype × sex × object interaction: F(2,102)=0.0985, P=0.9063 |
| Spatial test | Genotype, sex and object | 3-way ANONA, main effect of genotype: F(1,102)=3.618, P=0.06; main effect of sex: F(1,102)=0.0228, P=0.8803; main effect of object: F(2,102)=4.91, P=0.0092; genotype × sex interaction: F(1,102)=5.307, P=0.0233; genotype × object interaction: F(2,102)=1.422, P=0.246; sex × object interaction: F(2,102)=0.119, P=0.8879; genotype × sex × object interaction: F(2,102)=0.4905, P=0.6137 |
| Object test | Genotype, sex and object | 3-way ANONA, main effect of genotype: F(1,102)=0.5407, P=0.4638; main effect of sex: F(1,102)=0.0001, P=0.9704; main effect of object: F(2,102)=14.2, P<0.001; genotype × sex interaction: F(1,102)=2.504, P=0.1167; genotype × object interaction: F(2,102)=0.3414, P=0.7116; sex × object interaction: F(2,102)=0.1566, P=0.8553; genotype × sex × object interaction: F(2,102)=0.0584, P=0.9433 |

Supplementary table 2. Comparison of *Cul3* studies.

| Study | 2019, Rapanelli et al. | 2020, Dong et al. | 2021, Amar et al. | 2021, Morandell et al. | 2022, Xia et al. |
| --- | --- | --- | --- | --- | --- |
| Mouse Model | Emx1-Cre/Cul3^f/-^ Mice; Cul3^f/f^ Mice+ AAV-Cre(PFC) | GFAP-Cre/Cul3^f/-^ Mice | Cul3^+/-^ Mice (exon 6 insertion mutation via CRISPR) | Cul3^+/-^ Mice; Cag-CreER + TM/Cul3^f/-^ Mice; Emx1-Cre/Cul3^f/-^ Mice | Cul3^+/-^ Mice (Cul3^f/f^ mice crossed with Zp3-Cre Mice) |
| Western Blot | RhoA and Smyd3 increased in PFC; NR1 decreased; NR2A,NR2B unchanged | eIF4G1 increased in GFAP-Cre/Cul3^f/-^ brain, eIF4E, eIF4A1 unchanged; vGluT1, α/β-SNAP, NSF, VAMP1, PTEN, SHANK1 increased in GFAP-Cre/Cul3^f/-^ cortical neurons, PKA, SNAP25, Syntaxin 1, SYP, Synaptotagmin unchanged | RhoA and Active RhoA (RhoA GTP) increased in embryonic and adult cortex; Plastin 3 increased in embryonic cortex | Plastin 3 increased in Cul3^+/-^  mice brain and neural progenitor cells (NPCs), Smyd3, eIF4G1 unchanged. Cytoskeletal proteins Nisch, Plastin 3, INA increased in Emx1-Cre/Cul3^-/-^ cortex | RhoA, KCTD13 unchanged; Tropomyosin increased; Transgelin-2 decreased |
| Body Weight | Decreased body weight at P6; unchanged body weight at 5 weeks | Decreased body weight at P14 | Decreased body weight in adults | Decreased body weight at 0-10 weeks; unchanged body weight after 16 weeks | Decreased body weight in females at 13-15 weeks |
| Brain Size (MRI) | N/A | Decreased brain size at P14 | Decreased brain size in adults | N/A | N/A |
| Brain Morphology | N/A | Decreased cortical thickness only in GFAP-Cre/Cul3^-/-^ Mice at P14 | Unchanged cortical thickness, unchanged Sox2, Ki67 cell numbers in E14 Cortex | Unchanged cortical thickness in Cul3^+/-^ mice; neuronal migration defects and cell apoptosis, decreased Sox2 cells only in Emx1-Cre/Cul3^-/-^ E16.5 Mice cortex | N/A |
| Locomotion and Open Field | Increased total distance and velocity; unchanged time in center | Unchanged total distance and velocity; decreased time in center | Increased total distance and velocity; unchanged time in center | Unchanged total distance and velocity | Unchanged total distance and velocity; increased time in center and distance travelled in center |
| Hind Limb Clasping | N/A | N/A | N/A | Hind limb clasping in adult Cul3^+/-^ mice | N/A |
| Contextual Fear Conditioning | N/A | N/A | N/A | Normal fear acquisition and memory retention | N/A |
| Rotarod | Unchanged motor coordination | Unchanged motor coordination | N/A | Decreased motor coordination | Unchanged motor coordination |
| Hotplate sensitivity | N/A | N/A | N/A | N/A | Unchanged hotplate sensitivity |
| Olfactory Preference for Social Odors | N/A | N/A | N/A | Cul3^+/−^ mutant mice are hyper-reactive to the social odors | Unchanged preference for social odors |
| Elevated Plus Maze | N/A | Reduced time and entries in open arms; Unchanged time in closed arms or total distance | N/A | Unchanged ratio of time spent on open/open+closed arm | Unchanged time in open arms |
| Light/Dark Box | N/A | N/A | N/A | N/A | Unchanged time in light/dark sides |
| Prepulse Inhibition and Startle | Normal startle responses; reduced PPI at the highest stimulus intensity (85dB) in Cul3^f/−^ mice | N/A | Unchanged auditory startle responses | N/A | Unchanged startle responses and PPI |
| Grooming | Unchanged | Unchanged | Unchanged | N/A | Unchanged |
| Marble Burying | N/A | N/A | N/A | N/A | Unchanged |
| Reciprocal Social Interaction Task | N/A | N/A | N/A | N/A | Unchanged reciprocal social interaction |
| Three Chamber Social Test | Impaired social preference or "sociability" | Impaired social preference or "sociability"; Impaired social recognition memory | Normal social preference or "sociability" but less sniffing time on the social target; Impaired social recognition memory | Normal social preference or "sociability"; Impaired social recognition memory | N/A |
| Spatial and Novel Object Recognition | N/A | N/A | Reduced preference for novel object | N/A | Reduced spatial object recognition; unchanged novel object recognition |
| Sholl Analysis | Normal dendritic branching in PFC pyramidal neurons at 6 weeks | Normal dendritic branching in Hippocampus CA1 at P60 | N/A | Normal neuronal morphology in layer 2/3 pyramidal neurons in the somatosensory cortex of adult Cul3^+/-^ mice | Normal dendritic branching in hippocampal CA1 pyramidal neurons at 12 weeks |
| Dendrites | Unchanged number of dendrites, average dendritic length in PFC pyramidal neurons at 6 weeks | Normal dendritic branching in Hippocampus CA1 at P60 | Decreased number of dendrites, total dendritic length, unchanged soma area in Cul3^+/-^ primary cortical neurons at DIV14 | Unchanged number of dendrites, total dendritic length in layer 2/3 pyramidal neurons in the somatosensory cortex of adult Cul3^+/-^ mice | Slightly decreased dendritic branching in hippocampal CA1 pyramidal neurons at 12 weeks |
| Spine Density | Decreased basal spine density (decrease thin spines density) in PFC neurons at 6 weeks | Increased apical spine density and unchanged basal spine density in hippocampus CA1 pyramidal neurons at P60 | N/A | Unchanged spine density in layer 2/3 pyramidal neurons in somatosensory cortex of adult Cul3^+/-^ mice | unchanged apical spine density, only decreased stubby spine density in hippocampus CA1 pyramidal neurons at 12 weeks |
| mEPSCs | N/A | Increased frequency; unchanged amplitude in in CA1 pyramidal neurons of adult GFAP-Cre/Cul3^f/-^ mice | N/A | N/A | Increased frequency; unchanged amplitude in hippocampal CA1 neurons at 2-4 weeks |
| mIPSCs | N/A | Increased frequency; unchanged amplitude in in CA1 pyramidal neurons of adult GFAP-Cre/Cul3^f/-^ mice | N/A | N/A | N/A |
| sEPSCs | N/A | N/A | N/A | Decreased frequency and amplitude in layer 2/3 pyramidal neurons in somatosensory cortex of adult Cul3^+/-^ mice | N/A |
| sIPSCs | Unchanged frequency and amplitude in Cul3^f/f^ Mice+ AAV-Cre (STR) at 6 weeks | N/A | N/A | Unchanged frequency and amplitude in layer 2/3 pyramidal neurons in somatosensory cortex of adult Cul3^+/-^ mice | N/A |
| NMDA/AMPA Ratio | N/A | N/A | N/A | N/A | Unchanged in medial prefrontal cortex at 6-9 weeks |
| Input/Output Curve | Unchanged in NMDAR-EPSC and AMPAR-EPSC in frontal cortical pyramidal neuron at 6 weeks | N/A | N/A | N/A | Unchanged in evoked fEPSP slope versus stimulus intensity in hippocampus at 6-7 weeks |
| Paired Pulse Ratio (PPR) | Unchanged in Cul3^f/f^ Mice+ AAV-Cre (PFC) at 6 weeks | Reduced in CA1 pyramidal neurons of adult GFAP-Cre/Cul3^f/-^ mice | N/A | N/A | Unchanged in hippocampus at 6-7 weeks |
| LTP | N/A | N/A | N/A | N/A | Unchanged in hippocampus at 6-7 weeks |
| Proteomic Analysis | Emx1-Cre/Cul3^f/-^ Mice: 15 upregulated and 29 downregulated; Cul3^f/f^ Mice+ AAV-Cre (PFC): 72 upregulated and 93 downregulated; Cul3^f/f^ Mice+ AAV-Cre (STR): 37 upregulated and 11 downregulated; | GFAP-Cre/Cul3^f/-^ Mice (HET): 116 upregulated, 219 downregulated; GFAP-Cre/Cul3^f/f^ Mice (HOMO): 552 upregulated, 914 downregulated; | 736, 1239, and 1350 unique differentially expressed genes (DEG) in embryonic, early postnatal, and adult periods, respectively. | Cul3^+/−^ Mice embryonic cortex:31 upregulated, 33 downregulated; Emx1-Cre/Cul3^f/-^ Mice embryonic cortex:38 upregulated, 22 downregulated; Emx1-Cre/Cul3^f/-^ Mice pups: 146 upregulated, 94 downregulated | Cul3^+/−^ Mice: 63 upregulated, 70 downregulated |
| Validation of Proteomic Analysis Identified Proteins | Smyd3 increased | eIF4G1 increased. eIF4E, eIF4A1 unchanged | Plastin 3 increased | Plastin 3 increased. Smyd3, eIF4G1 unchanged. Cytoskeletal proteins Nisch, Plastin 3, INA increased | Tropomyosin increased. Transgelin-2 decreased |
